# Evaporation and transpiration processes and changes in their proportional relationships in cotton fields under varying degrees of film biodegradation

**DOI:** 10.3389/fpls.2025.1662751

**Published:** 2025-11-26

**Authors:** Hao Zhang, Dong Wang, Xun Zhang, Zhiyi Lv, Tao Jia, Haijun Liu, Yifan Wang, Tao Lin, Qiuxiang Tang

**Affiliations:** 1 College of Agriculture, Xinjiang Agricultural University, Xinjiang, China; 2 Xinjiang Jinfengyuan Seed Industry Co., LTD., Xinjiang, China; 3 Cotton Research Institute, Xinjiang Uyghur Autonomous Region Academy of Agricultural Sciences, Xinjiang, China; 4 Xinjiang Cotton Technology Innovation Center, Academy of Agricultural Sciences of the Xinjiang Uyghur Autonomous Region, Xinjiang, China; 5 Xinjiang Key Laboratory of Cotton Genetic Improvement and Intelligent Productions, Academy of Agricultural Sciences of the Xinjiang Uyghur Autonomous Region, Xinjiang, China; 6 National Cotton Engineering Technology Research Center, Academy of Agricultural Sciences of the Xinjiang Uyghur Autonomous Region, Xinjiang, China

**Keywords:** biodegradable film, rupture area, water consumption structure, evaporation volume, transpiration volume, yield

## Abstract

**Introduction:**

Biodegradable film (BE) is completely degraded during use, which effectively addresses the pollution caused by residual film in agricultural fields, serving as an environmentally friendly alternative to traditional polyethylene film (PE). However, BE surface degradation and rupture is a dynamic process, and the relationship between the coverage area and soil moisture and evapotranspiration partitioning changes has yet to be quantified.

**Methods:**

Therefore, a field experiment was conducted in 2021 and 2022, employing a film recognition method based on supervised classifiers to monitor the BE degradation area. Variations in evaporation and transpiration were determined in cotton fields under PE and BEs at different irrigation depths.

**Results:**

Under BE, for every 1% increase in film area damage compared to PE, the soil evaporation rate increased by 0.34 mm∺d^−1^ (R^2^ = 0.6027, n = 1613, and p = 0.028). The increased soil evaporation under BEs exacerbated the depletion of deep soil moisture. Compared to seedling stage, the soil water content at 60–80 cm depth under BEs decreased by 28.5–42.13% at the boll-opening stage. Increasing the irrigation depth enhanced the soil moisture content by 5.29–15.37%. Changing the irrigation depth promoted canopy development in cotton fields, increasing the leaf area index by 15.26–25.14% and plant transpiration by 10.32–17.86%.

**Discussion:**

Increases in irrigation depth and canopy coverage in cotton fields had similar inhibitory effects on evaporation as film application, mitigating the gradual increase in soil evaporation caused by BE rupture. Therefore, we suggest that irrigation quotas be appropriately increased when using BEs in oasis cotton areas to achieve coverage effects comparable to PEs.

## 1 Introduction

Based on the four functions of “temperature increase, moisture preservation, salt suppression, and grass suppression,” mulching technology has mitigated problems encountered in cotton planting in Xinjiang, such as recurrent low temperatures and high winds in the spring, seasonal unevenness resulting in water shortages, and heavy soil salinization. Thus, mulching has been widely adopted across the cotton-growing regions of Xinjiang (Qi et al., 2021; Zong et al., 2021). Nevertheless, current technologies for plastic film recovery are insufficient to fully eliminate the conventional plastic mulch used in agricultural fields (He et al., 2018), leading to the persistent accumulation of considerable plastic debris in the soil plow layer. This adversely affects cotton yield development and poses a threat of irreversible harm to farmland ecosystems (Gao et al., 2019). Thus, environmentally benign biodegradable mulch films have emerged as a promising alternative (Liu et al., 2022). These materials are predominantly fabricated from bio-based polymers, such as polylactic acid (PLA) and polyhydroxyalkanoates (PHA), and natural macromolecules, such as starch and cellulose. They are designed to progressively decompose into CO_2_, H_2_O, and biomass (Brodhagen et al., 2015; De Sadeleer and Woodhouse, 2024), preventing the long-term accumulation of plastic fragments in the soil (Touchaleaume et al., 2016; Yin et al., 2019). However, a significant challenge remains, as the mechanical strength and degradation of such biodegradable mulch films often fall short of agricultural requirements. To enhance both their degradability and physical performance, certain products incorporate photosensitizers and polyethylene as additives (Song et al., 2024). Although this approach can partially improve film functionality, it introduces new concerns, including inconsistent degradation performance and decomposition outcomes. These uncertainties impede the accurate assessment of field-scale water consumption under film mulching, which complicates irrigation management and water resource planning.

Extensive studies have been conducted on crop growth and water consumption in fields covered by biodegradable films (Kasirajan and Ngouajio, 2012; Deng et al., 2019). Although biodegradable films are comparable to traditional plastic mulches in maintaining crop growth, yield, and quality, their water retention capabilities remain relatively weak, particularly after degradation (Chen et al., 2021a; Zhao et al., 2023). Mulch functions to obstruct the exchange of water vapor between the soil and atmosphere, reducing ineffective evaporation and enhancing the soil moisture content (Zribi et al., 2015; Sharma et al., 2017; Qin et al., 2018). However, as biodegradable films gradually degrade over time, their coverage area decreases throughout the crop growth period, making it difficult to accurately monitor fluctuations in field evapotranspiration (ET) and water storage (Gu et al., 2021). Drip irrigation serves as a key technical measure for supplementing crop water requirements (Li et al., 2024). Research has shown that increasing the irrigation volume maintains soil moisture in a more uniform and suitable range (Lu et al., 2025). This meets the crop’s water uptake and transpiration demands. Although there is no significant advantage in water productivity, both crop yield and economic benefits are significantly improved. However, excessive irrigation provides a more abundant “source” for soil ET, increasing the total field ET (Guo and Li, 2024; Li et al., 2024).

ET in agricultural fields is comprised of crop transpiration and inter-canopy evaporation, representing a crucial element of the surface water and thermal cycle (Fan et al., 2017; Unkovich et al., 2018). Accurately distinguishing between these processes aids in elucidating the physiological water consumption of crops and ineffective soil evaporation, providing a theoretical basis for optimizing irrigation practices (Ding et al., 2013; Campbell et al., 2015; Hou et al., 2022). The primary methods for partitioning ET include the stem flow method (Campbell et al., 2015; Ham et al., 1990), eddy covariance technique (Scott, 2010), remote sensing methods (French et al., 2018), and the Bowen ratio energy balance approach (Irmak et al., 2014; Wu et al., 2025). However, these techniques often face challenges, such as high costs, technical complexity, and susceptibility to environmental interference. In contrast, lysimeters have notable advantages, including ease of operation, precise measurements, and environmental friendliness, and have been widely adopted for the direct measurement of soil evaporation (Evett et al., 1995; Oberholzer et al., 2017; Wang et al., 2022). Numerous studies have demonstrated that the results obtained from lysimeters are consistent with those derived from other methods (Ding et al., 2013; Zribi et al., 2015; Qin et al., 2018; Bian et al., 2024; Xia et al., 2024; Wei et al., 2025).

In agricultural production in arid and semi-arid regions, the changing biodegradable mulch breakdown area during degradation directly affects soil water conservation and ET. The irrigation amount is a key factor regulating field water conditions, further increasing the complexity of the ET system. Under different irrigation levels, the effects of mulch breakdown on the spatiotemporal distribution of soil moisture, total ET, and the ratio of its components differ significantly. This study aimed to accurately investigate the characteristics of ET and changes in its component ratios under the coupled effects of varying breakdown areas and irrigation levels in farmland with biodegradable mulch. A multi-classifier-based recognition method was used to monitor the dynamic mulch degradation area. The water balance method was applied to determine ET under different irrigation depths. The objectives were as follows: (1) to reveal the spatiotemporal characteristics of soil moisture in cotton fields covered with biodegradable mulch under different irrigation depths; (2) to quantify the dynamic changes in ET, the proportion of crop transpiration, and the proportion of soil evaporation in these cotton fields; and (3) to clarify the composition and transformation processes of ET under the coupled conditions of different irrigation depths and different mulch breakdown areas. This study provides theoretical support for synergistically managing biodegradable mulch application and water-saving irrigation in cotton fields in arid and semi-arid regions.

## 2 Materials and methods

### 2.1 Study site

**Figure 1:**
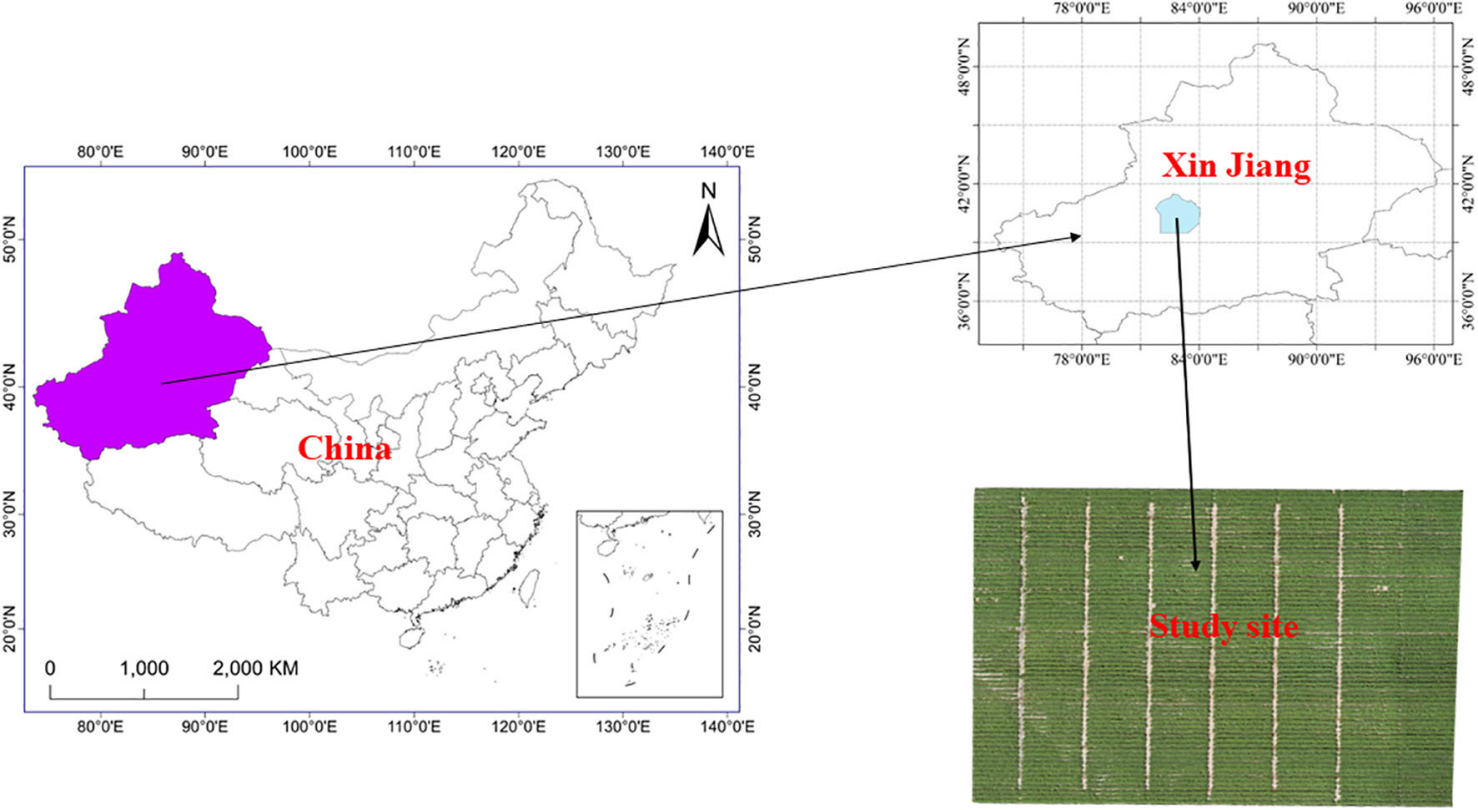
Experimental area and field layout. Located in Hailou Town, Shaya County, Aksu Region, Xinjiang, China. (N 41°17’, E 82°42’, 897 m above sea level).

**Figure 2:**
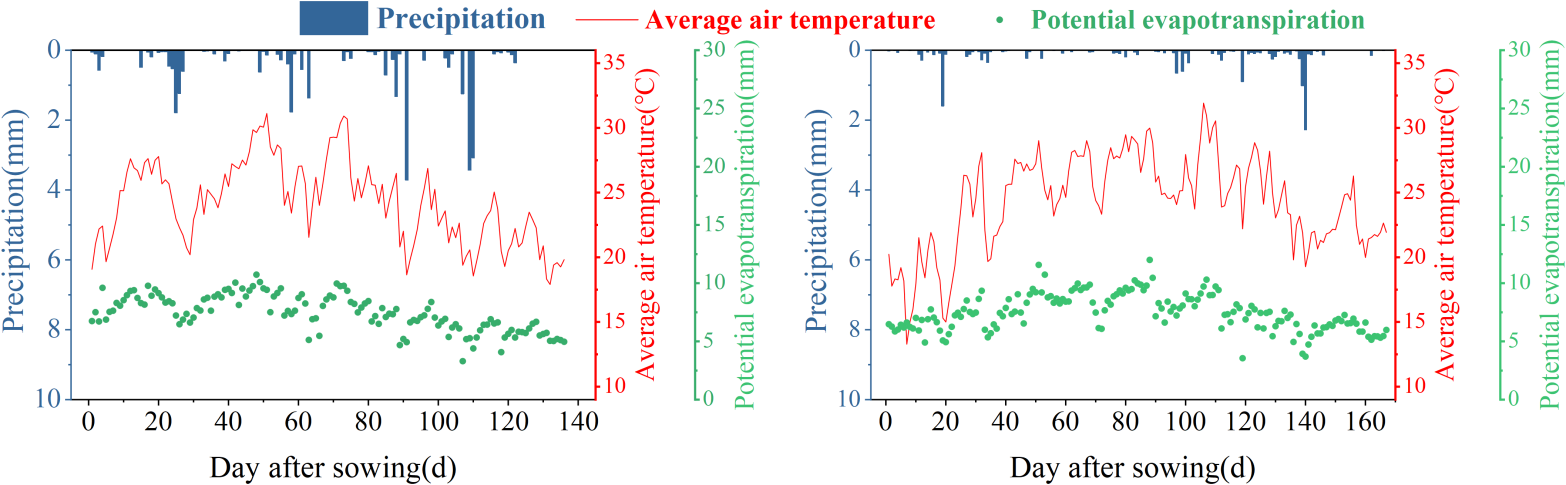
Changes in temperature and rainfall during the cotton growth period in 2021–2022.

A 2-year field experiment was conducted in Hailou Town, Shaya County, Aksu Region, Xinjiang (41°17’ N, 82°42’ E, 897 m above sea level, **Figure 1**) from 2021 to 2022. The variations in daily average air temperature, daily precipitation, and daily ET in the experimental area during the cotton growth period are depicted in **Figure 2**. In 2021, the average residual amount of plastic mulch in the cotton fields of this region was 216.0 kg·ha^−1^, which is 2.88 times the threshold limit for agricultural plastic residue set by China. The basic physicochemical properties of the soil in a 0–80 cm depth range at the experimental site are presented in **Table 1**. The groundwater level in the experimental area is greater than 5 m, making it unable to replenish the crop root zone, with upward supply considered negligible.

**Table 1:** The fundamental physicochemical properties of the soil in the experimental field prior to cotton sowing.

Indicator	2021 year	2022 year
Soil type	sandy loam	
Bulk density (g·cm^−3^)	1.50	1.48
pH	8.1	7.62
organic matter content (g·kg^−1^)	9.57	5.51
total nitrogen (g·kg^−1^)	0.53	0.38
alkali-hydrolyzed nitrogen (mg·kg^−1^)	39.35	49.63
available phosphorus (mg·kg^−1^)	17.98	8.00
available potassium (mg·kg^−1^)	111.82	109.47

### 2.2 Experimental design

A randomized block experimental design was employed, with plastic mulch as the main factor and irrigation depth as the sub-factor. The trial utilized four types of plastic mulch, as detailed in **Table 2**. Additionally, the reference ET (ET0) for the local cotton growing period was calculated to be 495 mm, based on the Food and Agriculture Organization-recommended Penman–Monteith formula (Allen et al., 1998). Consequently, three irrigation depths were established and evaluated in each type of mulch: W1: 315 mm (63.6% ET0); W2: 405 mm (81.8% ET0); and W3: 495 mm (100% ET0). In total, the experimental design yielded 12 treatments, each replicated 3 times, resulting in a cumulative total of 36 experimental plots. Each plot occupied an area of 64.98 m2 (length 9.50 m, width 6.84 m).

**Table 2:** Mulching film data.

Type of mulching film	Treatments	Raw material	Width/m	Thickness/mm	Color	Induction period/d
Traditional polyethylene mulch	PE	polythene	2.05	0.01	transparent	No
Fully biodegradable mulch	B1	PBS and PBAT	2.05	0.01	transparent	100
Thermo-oxygen-biodegradable mulch	B2	polythene and biodegradation additives	2.05	0.01	transparent	100
Fully biodegradable mulch	B3	PBS and PBAT	2.05	0.01	black	100

PBS represents polybutylene succinate; PBA represents poly (butylene adipate)/terephthalate.

The Penman-Monteith formula is as follows (Allen et al., 1998):


$${\text{ET}}_{0}=\frac{0.408\text{Δ}({\text{R}}_{\text{n}}-\text{G})+\text{γ}\frac{900}{T_{\alpha }+273}{\text{μ}}_{2}({\text{e}}_{\text{s}}-{\text{e}}_{\text{a}})}{\text{Δ}+\text{γ}(1+0.34{\text{μ}}_{2})}$$

where Δ is the slope of the saturation vapor pressure function (kPa·°C^-1^); R_n_ is net radiation (MJ·m^-2^·d^-1^); G is soil heat flux density (MJ·m^-2^·d^-1^); $T_{\alpha }$ is mean air temperature (°C); e_s_ and e_a_ are actual and saturation vapor pressure respectively (kPa); $\mu_{2}$is wind speed (m·s^-1^); γ is psychometric constant (kPa·°C^-1^).

The tested variety was locally recommended cotton cultivar ‘J206-5’, and a machine-harvested planting pattern of 66 + 10 cm was utilized, with a planting density of 26.50 × 104 plants·ha^−1^. The basal fertilizer application included nitrogen (N) at 162 kg·ha^−1^, P2O5 at 180 kg·ha^−1^, and K2O at 81 kg·ha^−1^. The initial plastic mulch coverage was set at 80%, and during the cotton growth period, irrigation and fertilization were carried out using a drip system beneath the mulch, with specific arrangements, as outlined in **Table 3**. Other management practices were consistent with standard field protocols.

**Table 3:** Irrigation and fertilization arrangements for Cotton.

Irrigation date		Irrigation depth (mm)			Topdressing quantity (kg·ha^-1^)		
2021	2022	W1	W2	W3	N	P_2_O_5_	K_2_O
June 23	June 15	31.5	40.5	49.5	22.164	7.320	4.500
June 30	June 22	31.5	40.5	49.5	22.164	7.320	4.500
July 07	June 29	31.5	40.5	49.5	29.544	9.720	4.500
July 14	July 06	31.5	40.5	49.5	29.544	9.720	5.600
July 21	July 13	31.5	40.5	49.5	30.264	13.320	5.600
July 28	July 20	31.5	40.5	49.5	37.164	13.320	5.600
August 04	July 27	31.5	40.5	49.5	36.444	9.720	6.700
August 11	August 03	31.5	40.5	49.5	36.444	9.720	6.700
August 18	August 10	31.5	40.5	49.5	29.544	9.720	6.700
August 25	August 17	31.5	40.5	49.5	22.164	7.320	5.600
Total irrigation depth (mm)		315	405	495			
Total topdressing quantity (kg·ha^-1^)					295.440	97.200	56.000

MAP is Monoammonium phosphate; PS is Potassium sulfate.

### 2.3 Data acquisition

#### 2.3.1 Monitoring the rupture area of biodegradable films

**Figure 3:**
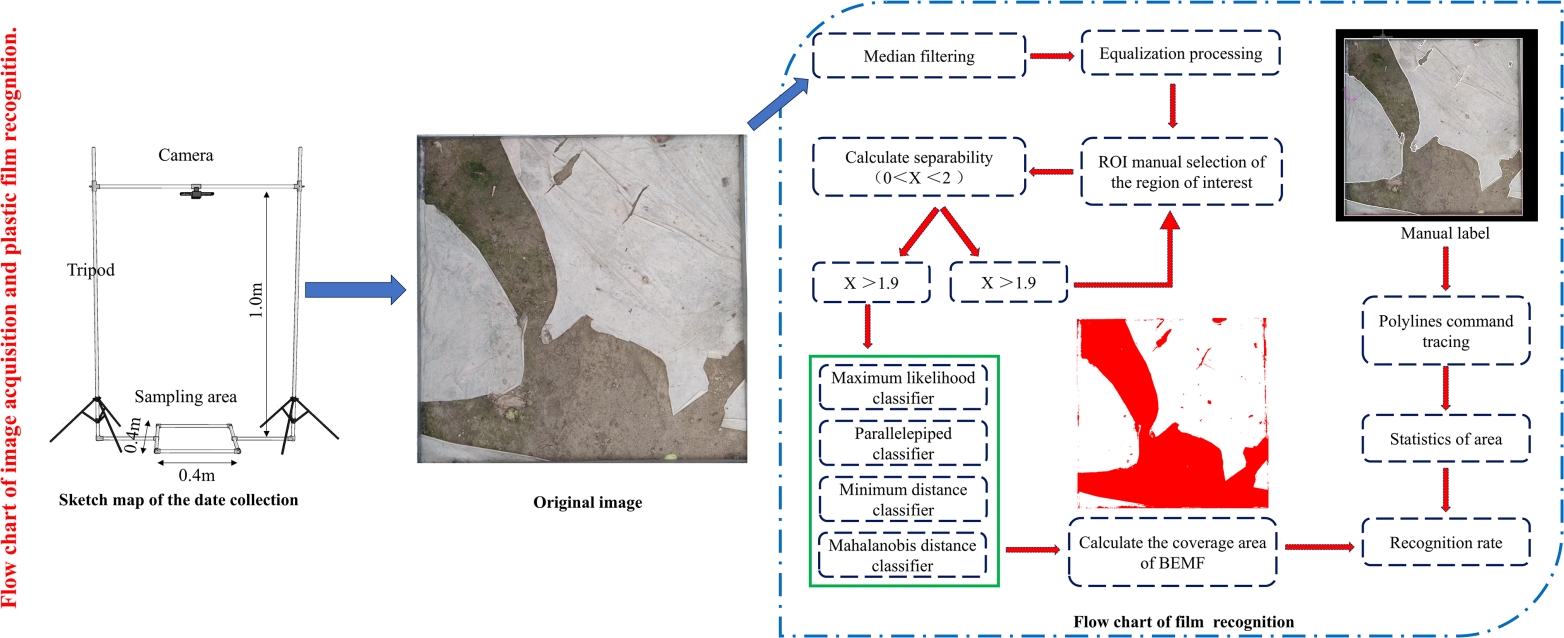
Flow chart of plastic film residual recognition.

To avoid external interference that may cause normal changes in the film, observations were conducted every 7 days following film application. Three observation points were selected in each plot to document biodegradable film degradation through photography. The camera was mounted on a fixed stand, ensuring a vertically downward orientation, as shown in **Figure 3**. The shooting distance was set at 1.0 m, and the sampling area post-capture measured 0.16 m^2^ (0.4 × 0.4 m).

**Figure 4:**
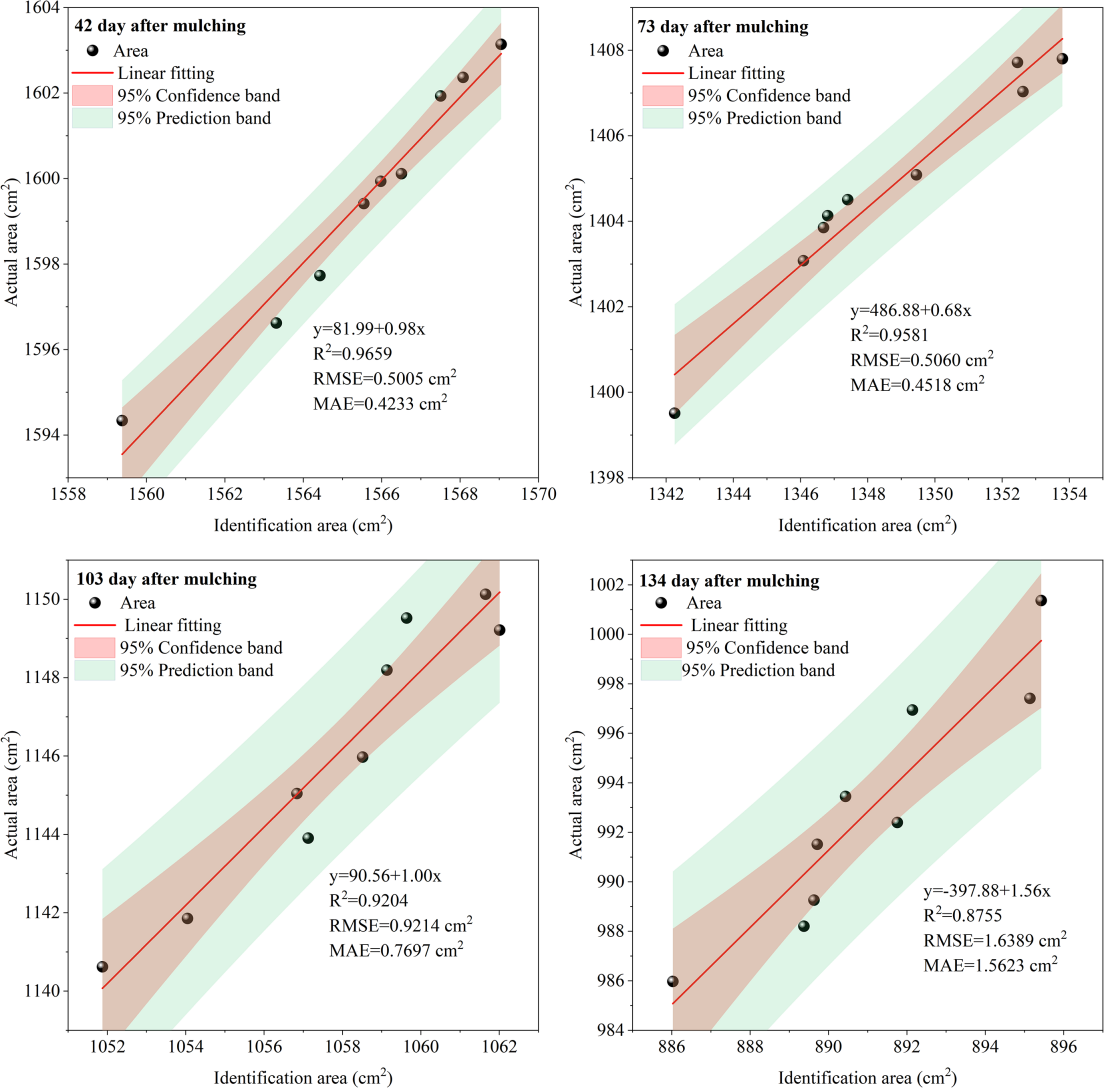
Recognition accuracy of biodegradable plastic film at different stages.

A method for monitoring the biodegradable film area based on supervised classifiers was employed for film identification, as illustrated in **Figure 3**. The main steps were as follows. (1) Image processing: The images were normalized and subjected to 3 × 3 median filtering to eliminate anomalies and histogram equalization to enhance the contrast of the objects in the images. (2) Film identification: The primary features within the sampling area included the film, soil, and drip irrigation tape. Regions of interest (ROIs) were delineated utilizing ENVI 5.3 software, and the areas covered by the film were manually selected to extract the reflectance information of the ROI. The reflectance characteristics of the extracted film-covered areas were analyzed using four classifiers based on traditional statistical analysis from the supervised classification module in ENVI (parallelepiped, minimum distance, Mahalanobis distance, and maximum likelihood classifiers), with the maximum likelihood and minimum distance classifiers demonstrating the best classification performance. The reflectance pixel points of the film-covered regions in the images were separated, with different colors used to distinguish between the ROIs and regions that were not of interest, thereby determining the film identification area in the sampling zone. (3) Accuracy verification: The images were imported into AutoCAD 2020 (Autodesk, Inc.) and scaled to dimensions of 0.4 × 0.4 m. The polyline command was then used to outline the damaged areas, forming closed-loop regions complemented by an area statistical command to individually identify the damaged areas in the study zone. The proportion of actual film damage was calculated by dividing the damaged area by the observed area (Chen et al., 2021b). Biodegradable film degradation was determined at different stages, with statistics on the identified area of film, actual area, and identification rate recorded, as shown in **Figure 4**. The results showed that with prolonged coverage duration, the identification rate of the film gradually diminished due to the film’s color shifting to yellow over time, blending with the soil’s grayscale, and reducing the identification rate.

The biodegradable film coverage rate in the sampling area (F) was calculated as follows (Chen et al., 2021b):


$$\text{F}={\text{S}}_{1}/{\text{S}}_{2}$$

The biodegradable film surface coverage rate at the field scale (Fm) was calculated as follows:


$$\text{Fm}=({\text{S}}_{\text{a}}\times \text{F})/{\text{S}}_{\text{b}}$$

where S1 represents the biodegradable film residual area in the sampling zone; S2 denotes the sampling region area; Sa indicates the total area covered by the biodegradable film prior to degradation; and Sb refers to the total area of the field.

The disintegration fraction (Fd) of biodegradable mulch under field conditions was calculated as follows:


Fd=1−Fm

#### 2.3.2 Monitoring of soil water content

**Figure 5:**
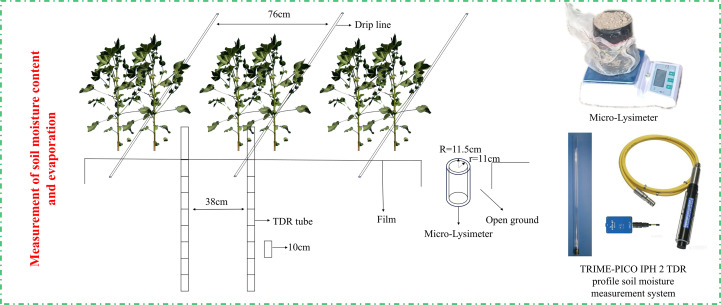
Cotton planting patterns and soil moisture measurement positioning points.

The soil water content was determined at 0–80 cm depth in each plot throughout the cotton growth period using the TRIME-PICO-IPH TDR profiling soil water measurement system. Each plot was measured at two points: the wide row measurement was taken at the midpoint of the second film in each plot; the narrow row measurement was positioned directly beneath the corresponding narrow-row drip emitter (**Figure 5**). Measurements were conducted in 10-cm intervals, with 3 repetitions for each layer, and assessments were performed every 7 days and after irrigation and rainfall events.

The soil water consumption was calculated using the agricultural water balance method (Wang et al., 2025; Fernández et al., 2020):


Soil water storage capacity:W=h×ρ×θ×10/100

Water consumption in cotton fields:ΔW=P+I−ET−K−R−D

h: soil layer depth (cm); ρ: soil bulk density (g·cm^-3^); θ: soil gravimetric moisture content (%); P: precipitation (mm); I: irrigation (mm); ΔW: changes in soil water storage between the beginning and end of the cotton growing season (mm). K is the groundwater recharge amount during the period (mm), which can be neglected when the groundwater table depth is greater than 2.5 m (in this experiment, the groundwater table depth was below 5 m, resulting in no groundwater recharge). D is the deep percolation amount (mm); R is the surface runoff amount (mm). Drip irrigation under mulch was used during irrigation events. This method delivers water directly and slowly to the root zone of the cotton plants via drip tapes. The application rate is low and the water is supplied gradually, allowing sufficient time for soil absorption. Consequently, surface ponding or runoff is virtually non-existent, and water is unlikely to percolate below the root zone into deeper soil layers. Therefore, both D and R are considered negligible and are disregarded.

The simplified equation is as follows:


ΔW=P+I−ET

The calculation method for crop water productivity (WPc) (Fernández et al., 2020):


WPc=Y/ET

Where: Y represents the yield of cotton seed cotton (kg·ha^-1^); ET denotes the water consumption in the cotton field (mm).

#### 2.3.3 Determination of soil evaporation

This study considered the environmental characteristics and practical requirements of cotton fields, fabricating micro-lysimeters of identical specifications suitable for measuring inter-row evaporation in cotton fields. The structure, placement, and position of the micro-evaporator were the same as described by Ding et al. (2013), Chen et al. (2021b), and Fang et al. (2021). In the bare soil layer of the cotton field, three plots were randomly selected for each treatment, with three miniature evaporators installed in each, resulting in nine micro-evaporators. These evaporators were constructed from PVC pipes, comprising two open-ended inner and outer cylinders. The inner cylinder had a diameter of 11 cm, a wall thickness of 2 mm, and a height of 15 cm, and the outer cylinder had a diameter of 11.5 cm, a wall thickness of 2 mm, and a height of 15 cm, as illustrated in **Figure 5**. The installation procedure was performed as follows: first, the inner cylinder was vertically inserted into the soil until its edge was level with the ground surface. Then, the soil column was excavated and sealed at the bottom with mesh netting. Finally, it was positioned in the pre-secured outer cylinder, ensuring that both surfaces were flush with the ground. This design prevents soil leakage and facilitates normal moisture exchange with the surrounding soil. The weight of the micro-evaporators and the soil was recorded every afternoon at 19:00 using an electronic balance with an accuracy of 0.01 g, and the difference in mass over 2 consecutive days represented the evaporation volume for 1 day. The undisturbed soil within the micro-evaporators was replaced every 6 days, and soil columns were changed immediately after rainfall or irrigation. The evaporation rate per unit time between the plants was calculated using the following formula:


$$\text{E}=\Delta \text{m}/{\text{πr}}^{2}$$

where Δm represents the mass difference measured by the micro-lysimeter within a unit time (g); r denotes the radius of the inner cylinder of the micro-lysimeter (mm). A reduction of 9.5 g·d^−1^ in the soil sample within the micro-lysimeter corresponded to an evaporation rate of 1 mm·d^−1^.

At the whole cotton field scale, the average soil evaporation (Es) was computed by incorporating the weighted ground cover rate (Fm) (Ding et al., 2013; Qin et al., 2018).


Es=E(1−Fm)

#### 2.3.4 Determination of yield

The harvest measurements were recorded on October 5, 2021, and September 20, 2022, when the cotton field exhibited a fiber extrusion rate exceeding 80%. During harvest, three random plots measuring 2.27 × 2.93 m were selected from each experimental area, and the number of cotton rows, the number of plants per row, and the number of bolls were recorded to calculate the average number of bolls per plant. Additionally, 30 cotton plants were randomly sampled from each plot and divided into 3 parts: upper (1–3 fruit branches), middle (4–6 fruit branches), and lower (≥ 7 fruit branches). From each section, 30 bolls were collected, dried to a constant weight, and weighed to calculate the weight per boll. The cotton seed yield per unit area was then calculated based on the effective plant density, average number of bolls per plant, and boll weight (Han et al., 2024).


$$\text{Seed cotton yield}(\text{kg}\cdot {\text{ha}}^{-1})=\text{Effective plant density}(\text{Plants}\cdot {\text{ha}}^{-1})\times \text{Average number of bolls per plant}(\text{Bolls}\cdot {\text{Plant}}^{-1})\times \text{Weight per boll}(\text{kg}\cdot {\text{Boll}}^{-1})$$

### 2.4 Data analysis

Statistical data analysis was conducted using the least significant difference (LSD) method in SPSS v. 22.0 (SPSS Inc., Chicago, IL, USA) to identify significant differences (P < 0.05). Graphical representations were generated using SigmaPlot (version 12.5, Systat Software, Inc., USA).

## 3 Results

### 3.1 Rate of surface damage to biodegradable films

**Figure 6:**
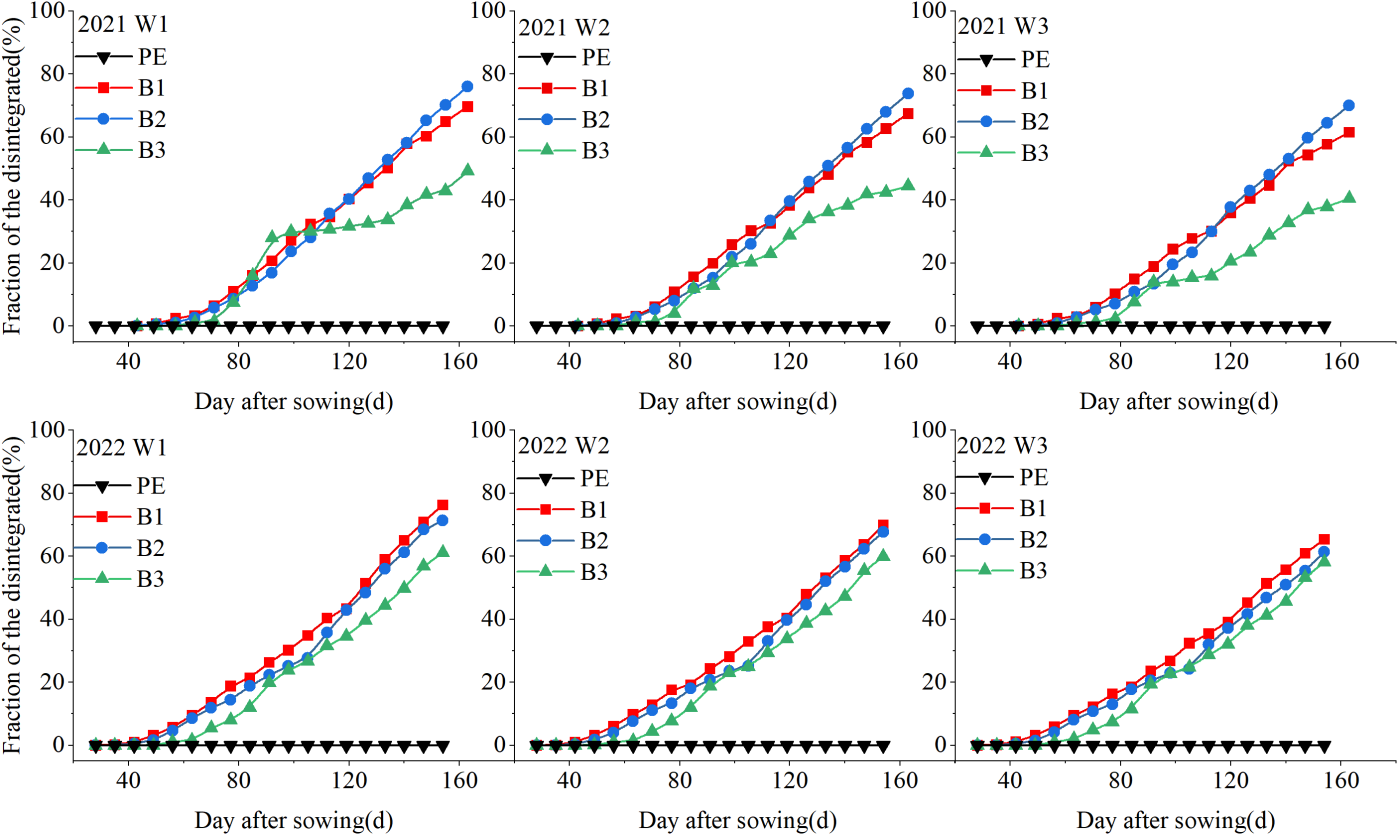
Proportion of membrane degradation area of mulch films under differing irrigation depths (2021–2022). W1: 315 mm (63.6% ET0); W2: 405 mm (81.8% ET0); and W3: 495 mm (100% ET0). PE: polyethylene mulch film; B1: Fully biodegradable mulch; B2: Thermo-oxygen-biodegradable mulch; B3: Fully biodegradable mulch.

Mulch film degradation and rupture in the field are illustrated in **Figure 6**. Notably, B2 and B1 exhibited the earliest formation of gaps, occurring at 28 and 42 days, respectively, approximately 7–14 days earlier than that in B3. After film degradation, the degradation rates of the three types of biodegradable film increased with the number of days after sowing, and the degradation rates of B2 and B1 were higher than those of B3 during the reproductive period. In 2021, the degradation rates of B2 and B1 reached 26.02 and 20.53%, respectively, at 60 days after sowing. B3 exhibited a degradation rate of 26.14% at 90 days after sowing, which was significantly lower than that of B2 and B1 by 20.78 and 10.19%, respectively. At 150 days after sowing, the degradation rates of the three films followed the order B2 > B1 > B3, with B2 achieving a degradation rate of 76.02%. Increasing the irrigation quota slowed the rupture speed of the biodegradable mulch. For example, in 2022, the degradation rate under W3 was 61.32% at 154 days after sowing, representing reductions of 9.34 and 14.01% compared to that under W2 and W1, respectively.

### 3.2 Leaf area index

**Figure 7:**
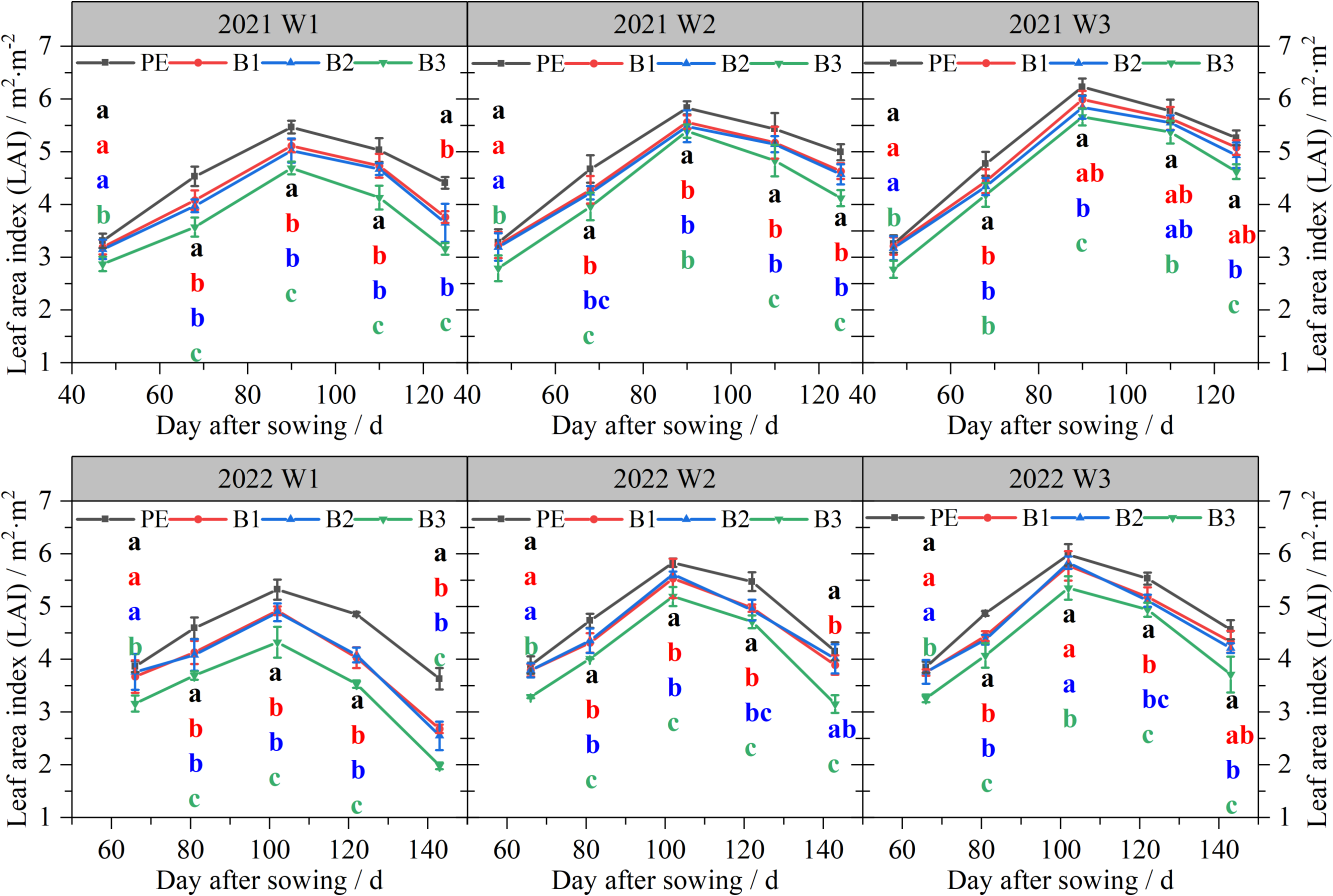
Dynamic changes in the LAI of cotton under different mulching types and irrigation depths in 2021 and 2022. W1: 315 mm (63.6% ET0); W2: 405 mm (81.8% ET0); and W3: 495 mm (100% ET0). PE: polyethylene mulch film; B1: Fully biodegradable mulch; B2: Thermo-oxygen-biodegradable mulch; B3: Fully biodegradable mulch. Different letters indicate significant differences at the 5% level.

As shown in **Figure 7**. the LAI for each treatment exhibited a trend of initial increase, followed by a subsequent decrease, peaking at 90–95 days after sowing in 2021a and 100–110 days in 2022a. Under W1, the peak LAI observed with PE was significantly (*P <* 0.05) higher than that with B2, B1, and B3, exceeding them by 6.44, 8.09, and 14.13%, respectively, in 2021, and by 7.52, 8.08, and 18.80%, respectively, in 2022. Moreover, the peak LAIs for B2, B1, and B3 were reached later. Increasing the irrigation volume increased the LAI under all four mulch films, and narrowed the gap in LAI among B2, B1, B3, and PE mulch films.

### 3.3 Soil water content

**Figure 8:**
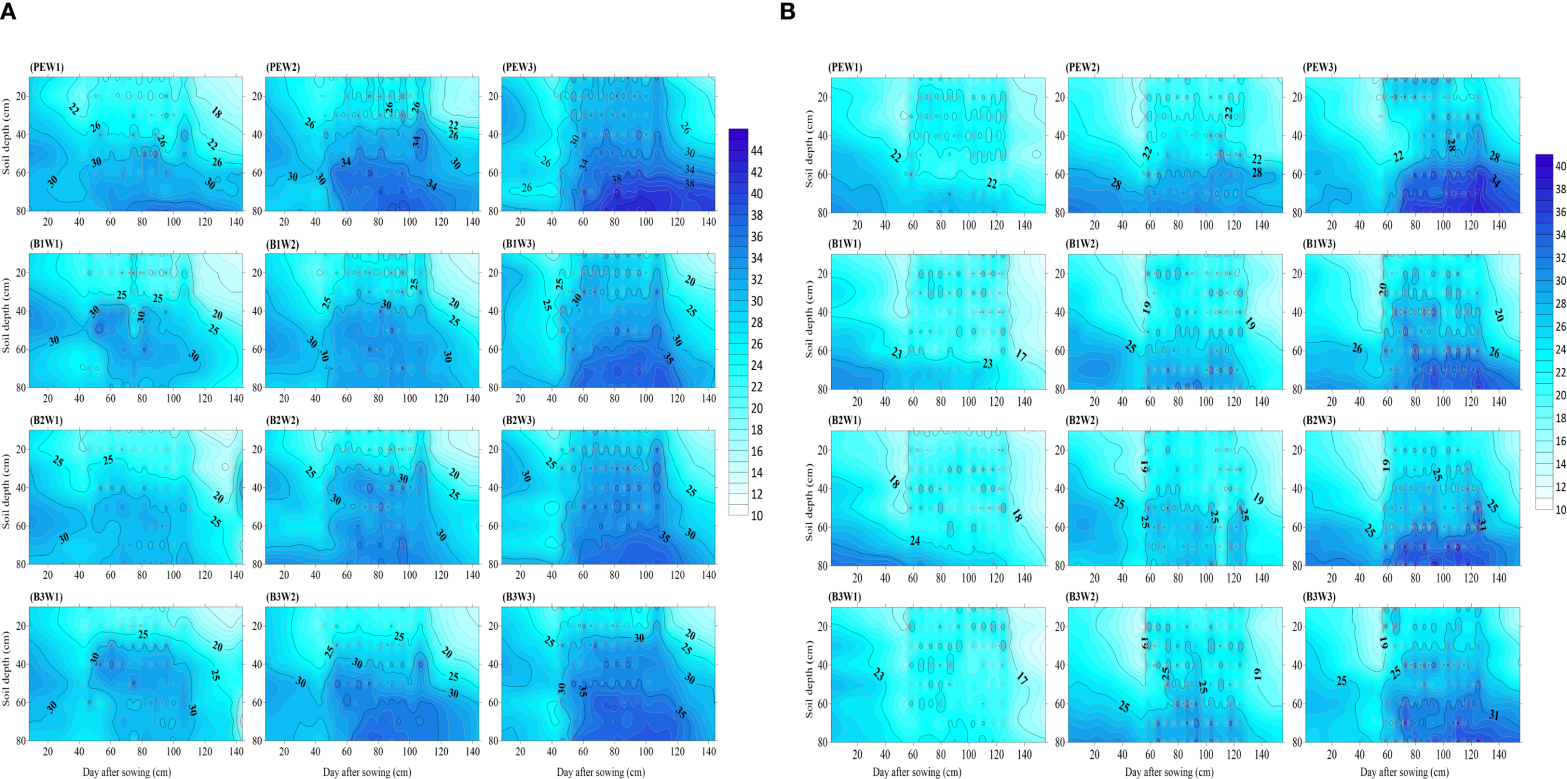
**(a)** Spatial and temporal dynamics of the soil moisture content at 0–80 cm under different mulching and irrigation treatments in 2021. W1: 315 mm (63.6% ET0); W2: 405 mm (81.8% ET0); and W3: 495 mm (100% ET0). PE: polyethylene mulch film; B1: Fully biodegradable mulch; B2: Thermo-oxygen-biodegradable mulch; B3: Fully biodegradable mulch. **(b)** Spatial and temporal dynamics of the soil moisture content at 0–80 cm under different mulching and irrigation treatments in 2022. W1: 315 mm (63.6% ET0); W2: 405 mm (81.8% ET0); and W3: 495 mm (100% ET0). PE: polyethylene mulch film; B1: Fully biodegradable mulch; B2: Thermo-oxygen-biodegradable mulch; B3: Fully biodegradable mulch.

The temporal and spatial variations of the soil moisture content at depths of 0–80 cm during critical cotton growth periods under different biodegradable films and irrigation depths from 2021–2022 are illustrated in **Figures 8a**, **b**. The changes observed over the 2 years consistently showed that the average soil moisture content increased with the irrigation depth across various treatments. Throughout the growth period, PE exhibited no degradation; in the 0–80 cm soil layer, the average soil moisture content under W3 was 5.57 and 1.92% higher compared to W1 and W2, respectively. In contrast, B2 demonstrated the most rapid degradation, resulting in a lower average soil moisture content under W1, W2, and W3 by 0.97, 3.34, and 4.39%, respectively, compared to that for PE, with no significant differences observed between B1 and B3. Analysis of the soil moisture content across growth periods showed that irrigation commenced at the budding stage, leading to the gradual downward infiltration of moisture into deeper soil layers. The average soil moisture content for B1, B2, and B3 under W1 was lower than that for PE by 1.59–5.95%. From the flowering to the boll-opening stage, mulch film degradation progressed in tandem with cotton growth, resulting in a gradual reduction of its moisture retention capacity; thus, the values for B1, B2, and B3 were lower than that for PE by 1.24–5.46% under W1–W3. Analyses of the soil moisture content in different soil layers revealed that in the 0–30 cm layer, the soil moisture content for B1, B2, and B3 under W1, W2, and W3 were reduced compared to PE by 3.29–6.78, 2.46–3.80, and 0.68–3.18%. In the 50–80 cm layer, the reductions compared to PE were 12.09–21.07, 10.14–13.05, and 5.31–8.50%.

### 3.4 Water consumption structure

#### 3.4.1 Water consumption

**Figure 9:**
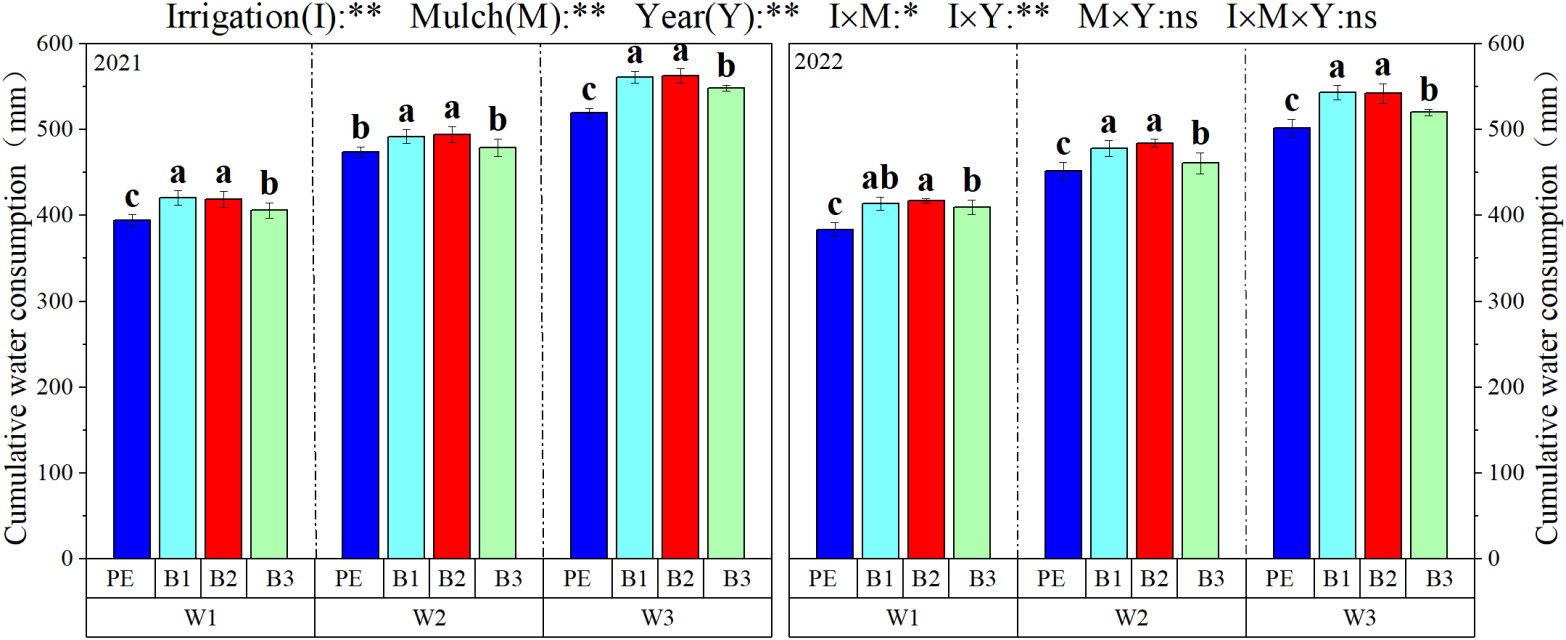
Water consumption changes in cotton fields in 2021 and 2022. W1: 315 mm (63.6% ET0); W2: 405 mm (81.8% ET0); and W3: 495 mm (100% ET0). PE: polyethylene mulch film; B1: Fully biodegradable mulch; B2: Thermo-oxygen-biodegradable mulch; B3: Fully biodegradable mulch. Different letters indicate significant differences at the 5% level. * and ** represent a significant difference at the 5 and 1% levels.

Water consumption was significantly influenced by irrigation (I), mulch (M), and year (Y) during each cotton growth stage. However, the interaction of I, M, and Y had no significant effect on water consumption throughout the growth period. The water consumption in cotton fields varied significantly due to differences in mulch coverage and irrigation depth. As shown in **Figure 9** and **Table 4**, as the growth process progressed, the water usage in cotton fields initially increased before subsequently decreasing for all treatments. In 2021, under W1, the water consumption for B2 and B1 increased by 8.75 and 9.66%, respectively, compared to PE, with significant differences observed. Under W2, differences in water consumption during the flowering and boll-opening stages were observed across treatments. During the flowering stage, the water consumption for B1 was the highest at 382.41 mm, significantly exceeding the PE treatment by 27.63 mm, whereas B3 recorded the lowest water usage at 349.58 mm, showing no significant difference from PE. Throughout the growth period, water consumption increased with greater irrigation depths; for instance, for PE, the total water consumption under W3 was higher than that under W1 and W2 by 6.56% (43.52 mm) and 13.07% (93.27 mm), respectively.

**Table 4:** Changes in water consumption and cotton in 4 growth stages under different mulch and irrigation treatments in 2021 and 2022.

Irrigation	Mulch	Water consumption (mm)							
		Seedling stage		Budding stage		Flowering-bolling stage		Boll opening stage	
		2021	2022	2021	2022	2021	2022	2021	2022
W1	PE	32.33a	27.56b	91.16a	66.67b	229.86c	234.41b	40.83c	55.08b
	B1	33.26a	30.06a	88.73b	71.65a	249.84a	238.37a	49.01a	73.92a
	B2	32.25a	30.58a	89.57ab	70.90a	247.96a	241.80a	49.37a	73.84a
	B3	33.30a	29.13a	84.18c	68.86ab	241.02b	240.92a	47.68b	70.73a
W2	PE	35.15a	29.22a	98.43a	69.68c	294.27c	288.71b	46.42c	64.14c
	B1	32.69a	30.29a	96.02a	73.07b	307.79a	299.37a	55.46a	75.37a
	B2	34.63a	30.80a	97.67a	76.89a	309.08a	302.17a	53.25ab	74.49a
	B3	34.00a	29.83a	92.99b	73.86b	299.53b	287.36b	52.51b	70.21b
W3	PE	36.75a	27.49a	96.08b	71.83b	333.27c	319.41c	53.34c	83.08c
	B1	37.72a	29.24a	104.97a	76.49a	350.61ab	339.37a	68.02a	98.16a
	B2	39.90a	30.94a	105.52a	76.88a	352.80a	341.17a	64.54b	93.47b
	B3	33.57b	29.95a	103.55a	72.86b	348.13b	327.14b	63.25b	90.32b
Source of variance									
Irrigation (I)		ns		**		**		**	
Mulch (M)		ns		**		**		**	
Year (Y)		*		**		**		**	
I×M		ns		**		**		**	
I×Y		ns		**		**		**	
M×Y		ns		**		*		**	
I×M×Y		ns		**		**		**	

W1: 315 mm (63.6% ET0); W2: 405 mm (81.8% ET0); and W3: 495 mm (100% ET0). PE: polyethylene mulch film; B1: Fully biodegradable mulch; B2: Thermo-oxygen-biodegradable mulch; B3: Fully biodegradable mulch. Different letters indicate significant differences at the 5% level. * and ** represent a significant difference at the 5 and 1% levels.

In 2022, under W1 during the flowering stage, the water consumption for B2 and B1 increased by 6.06 and 6.02%, respectively, compared to PE. Under W2, significant differences in water consumption were observed during the boll and flowering stages. Furthermore, under W3, B2 and B1 exhibited increases in water consumption of 7.24 and 11.51%, respectively, compared to PE. Water consumption increased with increasing irrigation depth during the reproductive period, with W3 surpassing W1 and W2 by 8.26 and 19.23%, respectively.

#### 3.4.2 The evaporation from the soil and the transpiration from the plants

**Figure 10:**
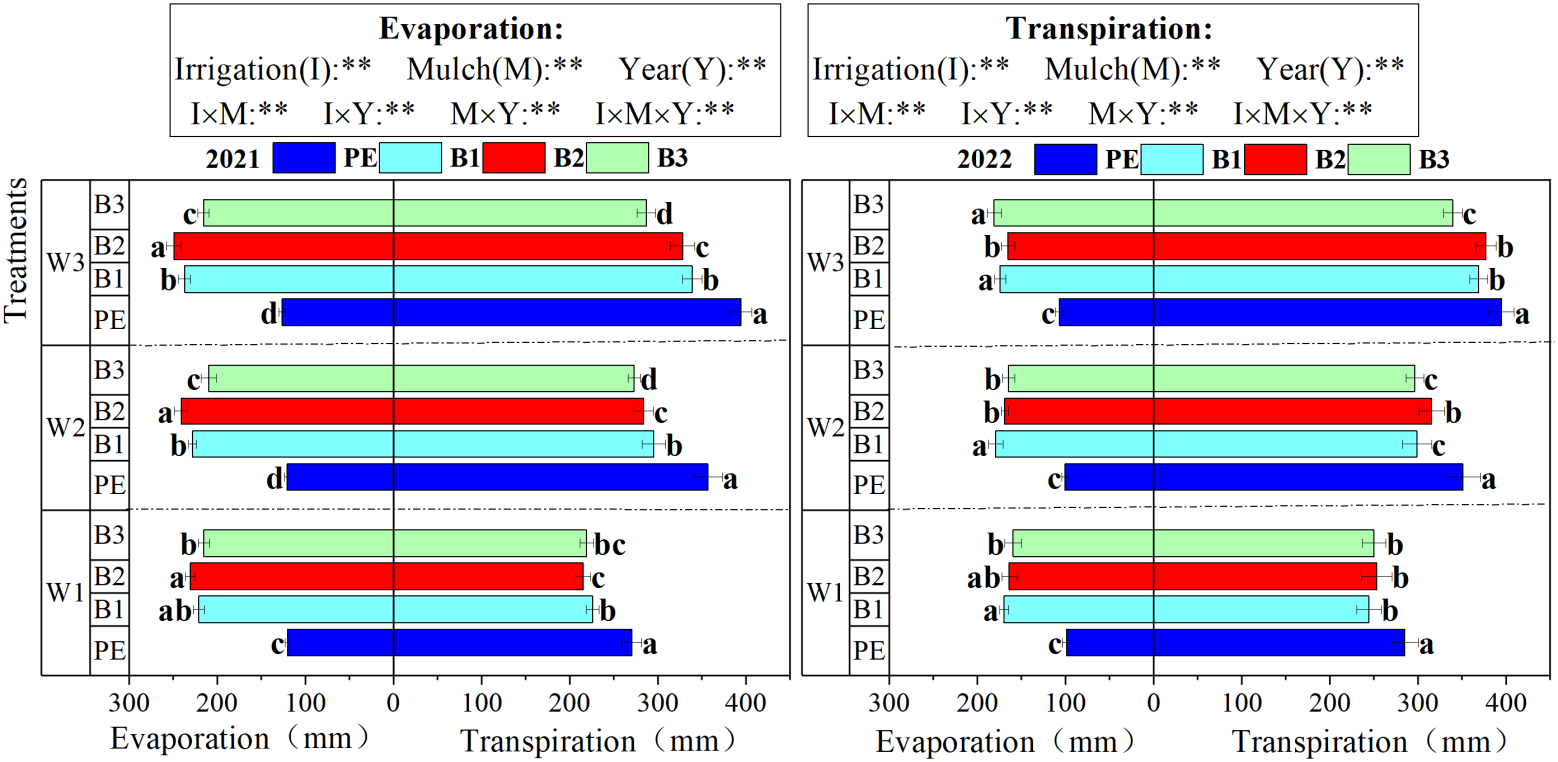
Changes of soil evaporation and plant transpiration at four main growth stages of cotton under different treatments. W1: 315 mm (63.6% ET0); W2: 405 mm (81.8% ET0); and W3: 495 mm (100% ET0). PE: polyethylene mulch film; B1: Fully biodegradable mulch; B2: Thermo-oxygen-biodegradable mulch; B3: Fully biodegradable mulch. Different letters indicate significant differences at the 5% level. ** represent a significant at the 1% levels.

Variations in soil evaporation and plant transpiration under different film coverings and irrigation depths in cotton fields are shown in **Figure 10**. Both evaporation and transpiration were significantly influenced by I, M, and Y, as well as their interactions. Over both years, soil evaporation significantly increased under biodegradable film coverage compared to PE coverage, whereas plant transpiration decreased under biodegradable films. For instance, in 2022, under W1, the evaporation and transpiration rates for PE were 99–285 mm, reflecting reductions of 38.15–41.89% and increases of 12.50–16.71%, respectively, compared to the three types of biodegradable films. Increasing the irrigation depth enhanced plant transpiration, although it did not significantly affect soil evaporation. For example, in 2022, for B1, the transpiration rate under W3 increased by 23.37 and 51.01% compared to W2 and W1, respectively.

**Figure 11:**
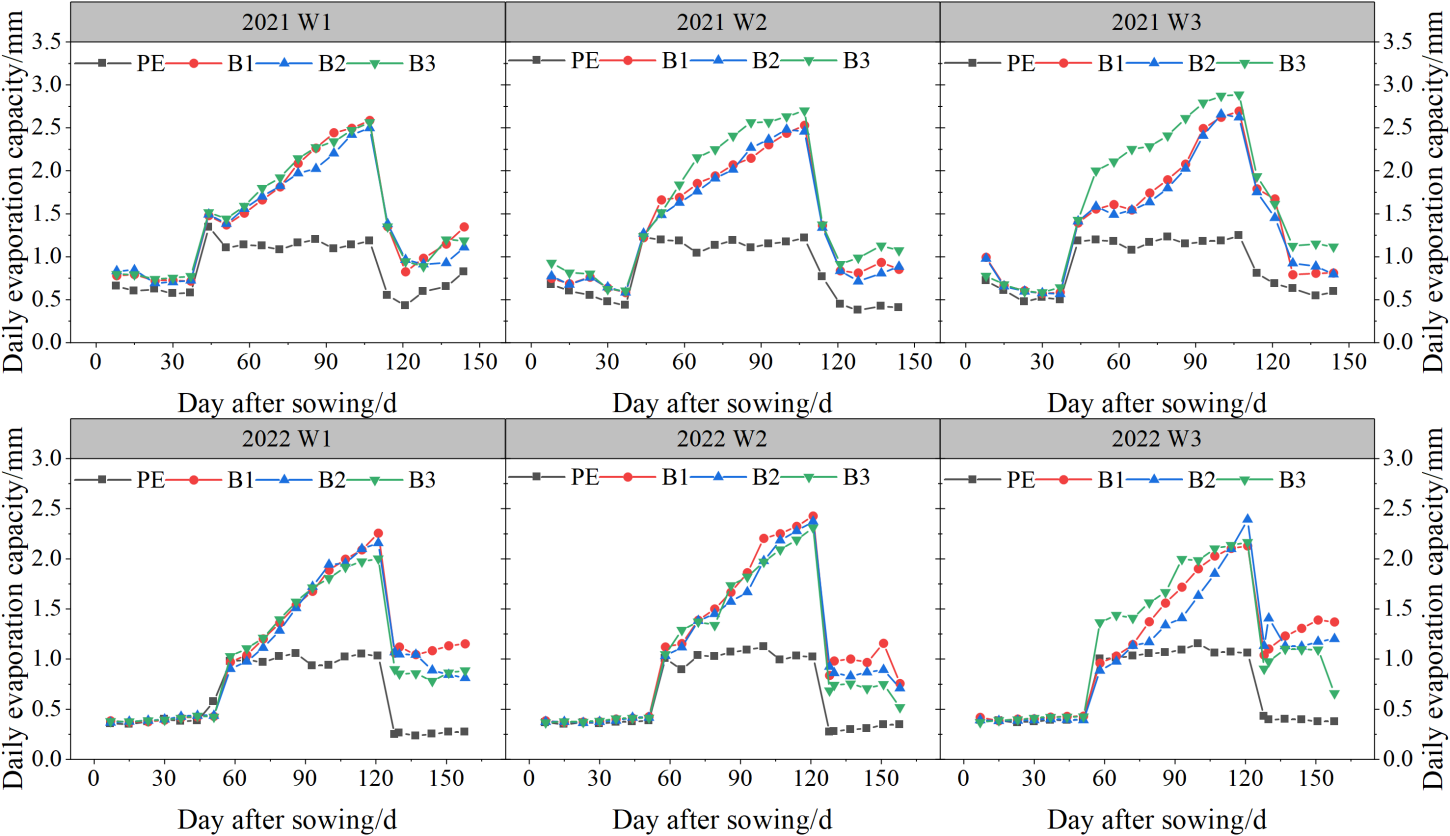
Stage changes of daily soil evaporation under different treatments. W1: 315 mm (63.6% ET0); W2: 405 mm (81.8% ET0); and W3: 495 mm (100% ET0). PE: polyethylene mulch film; B1: Fully biodegradable mulch; B2: Thermo-oxygen-biodegradable mulch; B3: Fully biodegradable mulch.

The impact of different mulch coverings and irrigation depths on inter-row evaporation in cotton fields during various growth stages is depicted in **Figure 11**. The inter-row soil evaporation throughout the cotton growth period followed a unimodal curve, demonstrating an initial increase, followed by a decrease as growth progressed. B2, B1, and B3 reached their peak values approximately 114 days post-sowing in 2021, and 128 days post-sowing in 2022, lagging behind PE by approximately 7 days. Under biodegradable mulch coverage, the daily soil evaporation increased with the expansion of the cracked area. Between 63–114 days post-sowing, the cracked areas of the biodegradable mulch increased from 9.54, 8.63, and 1.45% to 43.29, 42.80, and 34.54%, respectively. During this phase, the evaporation rates under biodegradable mulch coverage significantly increased, with daily evaporation for B2, B1, and B3 surpassing that for PE by 45.99, 50.76, and 41.18%, respectively.

### 3.5 Correlation analysis

**Figure 12:**
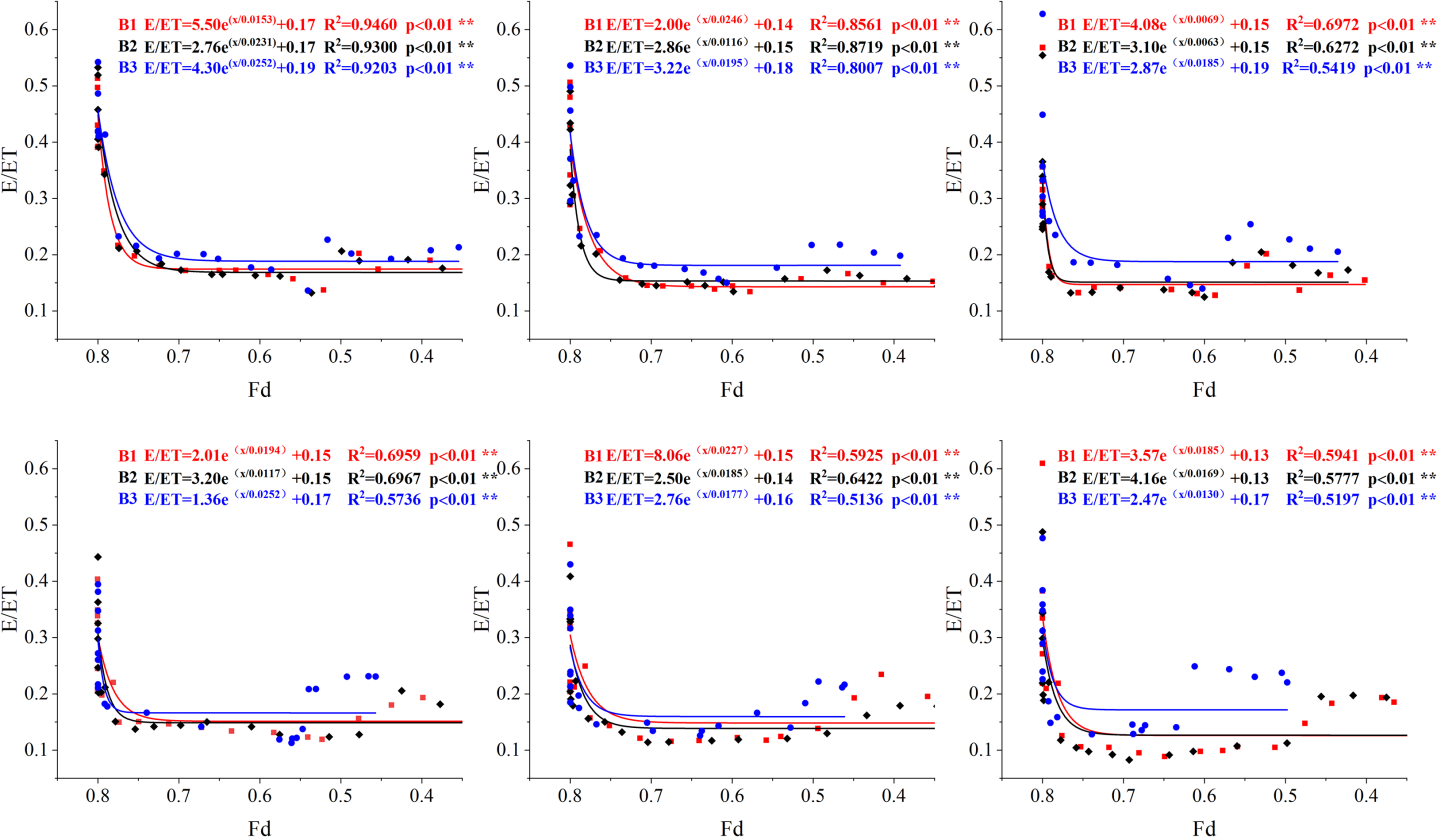
Dynamic relationship between E/ET and Fd (fraction of the disintegrated) under biodegradable mulching. W1: 315 mm (63.6% ET0); W2: 405 mm (81.8% ET0); and W3: 495 mm (100% ET0). PE: polyethylene mulch film; B1: Fully biodegradable mulch; B2: Thermo-oxygen-biodegradable mulch; B3: Fully biodegradable mulch. ** represent a significant at the 1% levels.

The relationship between the measured inter-row evaporation ratio (E/ET) and the coverage area ratio (Fd) is illustrated in **Figure 12**. As the area covered by the plastic film decreased, E/ET similarly decreased, following a power function trend. When the biodegradable film remained intact, with 0.7 ≤ Fd ≤ 0.8, E/ET decreased more significantly with the decline in Fd, resulting in a steeper slope in the E/ET curve. However, when the Fd fell below 0.7, E/ET decreased more gradually and remained relatively constant, becoming less sensitive to the increase in the area of film rupture. As the irrigation depth increased, the extent of decline in E/ET corresponding to a reduction in Fd became more pronounced, indicating that a deeper irrigation regime diminished the proportion of E to ET. Notably, after the flowering stage, inter-row evaporation was relatively higher under lower irrigation depths. This phenomenon was primarily attributed to the elevated soil moisture content resulting from deeper irrigation, which fosters cotton canopy growth and development, thereby producing an evaporative suppression effect, similar to that of the plastic film and mitigating the increase in soil evaporation due to biodegradable film rupture.

### 3.6 Yield and water use efficiency

As illustrated in **Table 5**, biodegradable mulch, irrigation depth, and their interaction had highly significant effects on seed cotton yield. The 2-year data showed that the seed cotton yield under biodegradable mulch cover showed a gradual increase with increasing irrigation depth, reaching a maximum under W3. The highest seed cotton yields for B1 and B2 covers were 5862 and 5872 kg·ha^−1^ in 2021 and 6789 and 6854 kg·ha^−1^ in 2022. Notably, these yields were not significantly different compared to the maximum yields obtained under PE cover.

**Table 5:** Effects of different degradation films and irrigation depth on yield and yield composition.

Irrigation amount	Film	Effective number of strains/×10^−4^·Plant		Single plant boll number/Boll·Plant^−1^			Single boll weight/g		Seed cotton yield/kg·ha^−1^		WPc/Kg·m^−3^	
		2021	2022	2021	2022	2021		2022	2021	2022	2021	2022
W1	PE	19.11a	22.57a	5.71a	4.65a	5.60a		5.91a	6096a	6201a	15.47a	16.16a
	B1	18.99a	22.62a	5.16b	4.37b	5.48b		5.72ab	5356b	5645b	12.73b	13.64b
	B2	19.76a	22.25a	5.29b	4.41b	5.16c		5.75a	5388b	5632b	12.86b	13.50b
	B3	19.34a	22.36a	5.29b	4.27b	5.07c		5.54b	5186c	5284c	12.77b	12.90c
W2	PE	18.59b	21.71b	5.70a	5.33a	5.55a		5.86a	5874a	6772a	12.39a	14.99a
	B1	19.20ab	23.12a	5.46ab	4.78b	5.20b		5.75ab	5450b	6345bc	11.08b	13.27b
	B2	19.34a	22.73ab	5.35b	4.96ab	5.35b		5.69b	5530ab	6399b	11.18b	13.21b
	B3	19.21ab	22.63ab	5.25b	4.74b	5.28b		5.66b	5317b	6068c	11.10b	13.16b
W3	PE	19.01a	21.89a	5.54a	4.93a	5.16c		6.10a	5434b	6575ab	10.46a	13.10a
	B1	18.99a	22.29a	5.81a	5.18a	5.32a		5.88b	5862a	6789ab	10.44a	12.50b
	B2	19.68a	22.25a	5.68a	5.04a	5.26b		6.11a	5872a	6854a	10.44a	12.64b
	B3	19.43a	21.91a	5.66a	4.98a	5.19c		5.97b	5699a	6514b	10.39a	12.52b
Source of variance												
Mulch(M)		*		**		**			**		**	
Irrigation (I)		ns		**		**			**		**	
Year(Y)		**		**		**			**		**	
M×I		ns		**		**			**		**	
M×Y		ns		ns		*			ns		**	
I×Y		ns		**		**			**		**	
M×I×Y		ns		ns		**			ns		*	

W1: 315 mm (63.6% ET0); W2: 405 mm (81.8% ET0); and W3: 495 mm (100% ET0). PE: polyethylene mulch film; B1: Fully biodegradable mulch; B2: Thermo-oxygen-biodegradable mulch; B3: Fully biodegradable mulch. WPc: crop water productivity. Different letters within a column and experimental year represent significant differences at P <0.05. * and ** represent a significant difference at the 5 and 1% levels. respectively; ns represents no significant difference at the 5% level.

Combining the yield components, the decrease in the number of bolls per plant and boll weight per boll was the main reason for the decrease in seed cotton yield with increasing irrigation under W1 and W2. For instance, in 2021, the number of bolls per plant and the weight of individual bolls for B2 cover under W1 were 5.29 Pro·Plant^−1^ and 5.16 g, representing reductions of 7.36 and 7.86%, respectively, compared to PE. However, WPc consistently decreased with increasing irrigation. WPc was lowest when the seed cotton yield was highest under B1 and B2.

## 4 Discussion

### 4.1 Evaluation methods to determine the degradation effects of biodegradable films

Currently, our nation primarily addresses agricultural residual film pollution through mechanical recovery and the use of biodegradable mulching films. The degradation performance of biodegradable films is a critical factor determining their potential for widespread application. However, variations in their chemical composition, production processes, and environmental conditions result in differing degradation characteristics, leading to uncertainties in crop yields (Chen et al., 2021a). Therefore, controlling the biodegradable mulch degradation process so that its degradation cycle matches the crop growth cycle has become an important technical challenge.

At present, most scholars rely on visual assessment to monitor biodegradable film degradation (Yin et al., 2019; Yang et al., 2023; Sellami et al., 2024; Wang et al., 2024); however, this method is influenced by subjective biases and has limited precision. Some researchers calculate the weight loss rate of films through manual collection or assess the disappearance area of the films by placing them on graph paper (Zhang et al., 2021), but this approach is time-consuming and labor-intensive, delaying outcomes that do not synchronize with the crop production process. Given its advantages of high spatial resolution, low cost, short cycle time, and high repeatability, unmanned aerial vehicle (UAV) low-altitude remote sensing technology has been widely used for crop growth monitoring (Zhao et al., 2019; Su et al., 2018; Li et al., 2022). In recent years, some scholars have also begun preliminary explorations into detecting agricultural film pollution. Yang et al. (2024) and colleagues evaluated a drone-based image segmentation algorithm for residual film and performed field tests for film identification, finding an R2 for UAV-evaluated film pollution and manual evaluation of 0.966. This study also validated the efficacy of the method, employing a color channel threshold segmentation algorithm to achieve precise identification and coverage detection of agricultural residual films. Using this approach, we successfully obtained data on the rate of degradation film damage every 7 days, with an identification accuracy exceeding 89%, indicating a high detection reliability. However, these results are susceptible to interference from contaminants on the film surface, such as dust and water stains. Research by scholars, such as Ha et al. (2020), based on the fusion of optical remote sensing images and radar imagery, has demonstrated that multi-source data fusion technology significantly improves mulch recognition compared to using a single data source. Therefore, subsequent studies should select more stable color components or other easily distinguishable feature variables. The automatic classification accuracy and anti-interference capability for residual mulch films of different colors can be systematically enhanced by incorporating multi-source data fusion technology.

### 4.2 The impact of biodegradable film coverage on moisture movement in cotton fields

The application of mulching technology effectively preserves soil moisture. A sealed space of 2–5 mm is formed between the film and the surface soil layer, obstructing the direct exchange of soil moisture with the atmosphere (Zhou et al., 2009; Liu et al., 2016). This spatial structure confines the water vapor generated by evaporation to a limited range, significantly enhancing the relative humidity of the air beneath the film and creating an internal moisture circulation system between the film and the soil surface. This fundamentally alters the open movement pattern of soil moisture under traditional uncovered conditions, suppressing moisture evaporation loss and enabling the topsoil to maintain a higher water content (Sharma and Bhardwaj, 2017; Qin et al., 2018). Furthermore, mulching facilitates the upward movement of deep soil moisture to the surface, which improves the utilization efficiency of deep water, significantly enhancing soil water consumption rates during critical periods of crop water demand (Gu et al., 2016). Our results indicate that the moisture-retaining effect of biodegradable films is inferior to that of conventional polyethylene films, with B3 exhibiting the lowest moisture content during the early growth stage. Irrigation begins in the mid-growth stage of cotton, coinciding with increasing temperatures and evaporation. Notably, the soil moisture content across all treatments began to vary significantly, with all three biodegradable films showing a lower soil moisture content under W1 and W2 compared to PE. In the later cotton growth stages, when the irrigation depth increased to W3, the soil moisture content beneath B1 approached that of PE, while the moisture content beneath B2 and B3 increased to varying degrees, but remained lower than that of PE and B1. This is attributed to the increased ineffective evaporation of soil moisture following film degradation, which diminishes their water retention capacity and subsequently reduces the soil moisture content. As the irrigation depth increases, robust plant growth leads to a denser canopy coverage, reducing soil evaporation and enhancing the soil moisture content.

The moisture content of the surface soil significant fluctuates due to the dual influences of plant root absorption and surface evaporation (Liebhard et al., 2022; Fang et al., 2022). During specific growth stages, crops experiencing water stress may benefit from moderate irrigation, which can induce a compensatory growth effect. However, excessive irrigation negates this compensatory effect and leads to adverse phenomena such as delayed maturation and excessive vegetative growth (Cui et al., 2015; Comas et al., 2019). Excessive irrigation may trigger excessive vegetative growth and delay maturity. These findings align with previous research, indicating that biodegradable film degradation begins during the cotton flowering stage, weakening its moisture retention capability and resulting in a lower soil moisture content compared to the control PE film. However, under W3, the soil moisture content in the 0–30 cm layer increased across all treatments, particularly for B1, which exhibited a 3.72% higher moisture content than the control PE film. This suggests that increasing the irrigation depth enhances the lateral movement of soil moisture beneath the biodegradable film, leading to moisture accumulation in the upper soil layers and reducing ineffective water, which facilitates efficient water use. Under PE mulch in 2022, the soil moisture content in the 50–80 cm layer at the W3 irrigation depth registered 33.55%, representing increases of 19.62% and 63.06% compared to W2 and W1, respectively. This may be because the non-degradable PE film enhanced its vertical moisture movement capacity with increased irrigation depth, which results in a higher moisture level in the lower soil layers. Contributing to excessive vegetative growth in cotton bolls and consequently reducing yield.

### 4.3 The impact of biodegradable film coverage on water consumption and crop water productivity in cotton fields

ET typically comprises soil evaporation and plant transpiration. Studies have shown that increased irrigation leads to a higher crop LAI, which enhances transpiration (Zuo et al., 2023). This indicates that the impact of irrigation on latent heat flux involves changes in soil evaporation and transpiration (Li et al., 2024). In arid and semi-arid regions, where drip irrigation under mulch is predominant, the effects of irrigation and mulching methods on ET changes warrant in-depth investigation. Irrigation influences farmland ET by affecting soil moisture (Guo and Li, 2024). When irrigation is insufficient, the soil moisture in the root zone drops below the crop’s critical threshold. Restricted stomatal conductance strongly suppresses transpiration, decreasing ET. Increasing the irrigation volume improves water conditions, resulting in a higher LAI. This makes transpiration the dominant component of ET and suppresses ineffective evaporation from surface soil through the shading effect of leaves (Zuo et al., 2023; Gong et al., 2024). This creates a synergistic effect of “enhanced transpiration and suppressed evaporation,” which aligns with the findings of this study. The present study observed that as irrigation depth increased, E/ET gradually decreased because additional irrigation compensated for water demand during the cotton flowering and boll stages, increasing plant LAI. However, a higher LAI did not increase soil water consumption. Due to inter-leaf shading, more surface soil was covered, inhibiting ineffective soil evaporation. These results indicate that appropriately increasing the irrigation depth can balance inter-row evaporation under biodegradable mulch, producing a suppression effect similar to that of PE mulch.

The application of mulching significantly enhances soil moisture conditions due to its physical barrier properties. Research has demonstrated that the impermeability of mulch suppresses soil moisture evaporation, maintaining a stable soil water content and exhibiting remarkable water retention effects (Sharma and Bhardwaj, 2017; Burato et al., 2025). During the early stages of coverage, the use of standard PE film can reduce soil evaporation by up to 90%. In a study conducted in the eastern Loess Plateau, Chai et al. (2022) confirmed that mulching significantly increased the soil moisture content, decreased soil evaporation, and enhanced plant transpiration compared to uncovered treatments. This moisture regulation effect displays distinct phase characteristics. In the early coverage stage, when the crop canopy has yet to develop, field water consumption is driven by soil evaporation. The physical barrier of the mulch disrupts vapor exchange between the soil and atmosphere, effectively curbing moisture loss (Deng et al., 2022). In the later coverage stages, as crops develop and canopy coverage increases, the interception of solar radiation by leaves reduces the energy reaching the soil surface, diminishing soil evaporation (Sadras et al., 1991). Despite enhanced transpiration from the crops, the overall reduction in water consumption remains limited. Some studies have shown that the water consumption intensity in mulched fields may surpass that of uncovered treatments (Tan et al., 2022), which is attributed to the warming effect of the mulch, which promotes crop growth. Although soil evaporation continues to be suppressed, vigorous plant transpiration increases water consumption overall. Our study showed that the increase in water consumption in cotton fields utilizing biodegradable films was primarily due to a significant increase in soil evaporation following biodegradable film rupture (increased significantly by 91.06 mm in 2021 and 67.47 mm in 2022), although transpiration under the biodegradable film decreased (with significant decreases of 66.53 mm in 2021 and 38.74 mm in 2022). Furthermore, this study revealed that as the irrigation depth increased, the disparity in the soil moisture content between cotton fields under B1 and those under PE gradually diminished, becoming statistically insignificant. This occurs because the increased irrigation depth compensates for the moisture demands in cotton during the flowering and boll-setting stages, promoting robust growth and maintaining a larger canopy and shaded area, which inhibits ineffective soil evaporation.

The process of vapor transfer and transformation beneath the mulch is relatively complex. Once water vapor condenses under the film, the subsequent diffusion of vapor into the atmosphere through secondary evaporation is difficult to measure using conventional methods. The vapor evaporating from film seams due to damage is also difficult to quantify. To estimate soil evaporation in mulched fields, studies have employed miniature evaporation devices. However, to estimate soil evaporation in agricultural fields, many studies have used a micro-evapotranspiration meter, but the evaporation process of soil under a membrane covering is complicated, as the evaporated water vapor adhering to the membrane forms condensed water, condensed water undergoes secondary evaporation, becoming water vapor, which diffuses through the membrane seam to the atmosphere. The use of a micro-evapotranspiration meter to determine the problem of evaporation in the soil under the membrane needs to be verified in further experiments.

As an efficient water-saving agricultural practice, mulching has been widely adopted in agricultural production. Studies have shown that the use of ground cover increases crop yields by 45.5%, on average, while increasing water use efficiency by 58.0% (Sun et al., 2020). Water is the primary limiting factor for crop growth and yield formation in arid and semi-arid regions (Isaev et al., 2021). Increasing irrigation promotes the accumulation of crop photosynthates and prevents the yield reduction caused by insufficient water supply. Although this enhances crop yield and economic benefits, water productivity does not improve significantly and may even decrease. However, excessive moisture can stimulate vigorous growth of the plant’s aboveground vegetative organs, reducing the root-to-crown ratio, leading to premature canopy closure and severe shedding of buds and fruit, and decreasing light and air permeability in the field, which reduces yield (Li et al., 2024). Appropriate water can coordinate cotton root and crown growth, maintain an appropriate root–crown ratio, improve dry matter accumulation, coordinate reproductive growth, promote the use of photosynthetic products in product organ operation and distribution, create conditions favorable for boll yield, and result in water-savings and high yield, shaping cotton for mechanical harvesting (Wang et al., 2019). The findings of this study align with those of previous research, indicating that B1 under W3 is more advantageous for the transport of photosynthetic assimilates to cotton bolls, increasing the number of bolls per plant and boll weight, which enhances yield. In contrast, under W1 and W2, insufficient water supply decreases the amount of photosynthetic products in cotton covered by B1, B2, and B3, slowing the growth rate of reproductive organs and significantly decreasing yields below those achieved with PE.

Furthermore, preliminary findings of this study demonstrate that although biodegradable mulch films involve higher production costs, they eliminate subsequent expenses associated with retrieval and disposal (Zhang et al., 2025). By applying an optimized irrigation depth of 495 mm, the yield risks potentially induced by premature degradation can be fully mitigated, thereby establishing economic feasibility. More importantly, from an environmental perspective, fully biodegradable mulch film (B1) fundamentally eliminates the long-term adverse effects of plastic residues on soil structure, microbial activity, and crop root development, thereby preventing irreversible damage to farmland ecosystems caused by “white pollution”. Hence, under the optimized W3 irrigation regime, the adoption of biodegradable mulch film represents a strategic approach that harmonizes short-term economic benefits with long-term environmental sustainability. However, the implementation of this strategy must take into account local water resources and their utilization efficiency. To that end, our subsequent research will focus on employing more precise irrigation scheduling to target limited water resources specifically during the mid-to-late growth stages, when cotton is most sensitive to water availability. The objective is to further reduce irrigation depth without compromising yield in cotton fields using biodegradable mulch, thereby addressing severe water scarcity challenges.

### 4.4 The perspectives and limitations of this study

As a novel agricultural covering material, biodegradable film possesses the advantages of traditional plastic mulches and mitigates the issue of residual pollution, establishing a foundation for its application in the agricultural sector (Wang et al., 2022). However, current information on the impacts of biodegradable mulch films and their derived microplastics and additives on agricultural ecosystems remains limited (Xiong et al., 2024). Many related mechanisms and conclusions remain uncertain. Its uncontrollable degradation characteristics pose a significant barrier to its widespread use (Liu et al., 2022). To address this challenge, researchers are investigating methods to regulate the degradation rate. For instance, by adjusting the formulation, structure, and processing techniques of the film, controllable degradation rates can be achieved under varying environmental conditions. This adaptability enables the selection of biodegradable films with suitable degradation rates tailored to different crop growth cycles and regional climatic characteristics, enhancing their efficacy and environmental compatibility. This approach necessitates extensive research and experimentation and requires substantial investment of time, resources, and manpower, which results in high development costs and poses commercial limitations on its application in agricultural production. Furthermore, the uncontrollable degradation process of biodegradable mulch results in a time gap between the physiological needs of crops and field management, reducing crop yields, which explains why it has not been popularized on a large scale.

Image segmentation involves the separation of distinct regions within an image based on specific criteria, enabling the extraction of ROIs. It serves as a critical preprocessing step for such tasks as image recognition, scene analysis, and object detection. Researchers have applied image processing techniques to mulch film identification. Ha et al. (2020) developed a robust remote sensing methodology for monitoring plastic mulch-covered croplands, utilizing spectral and textural features from Landsat-8 OLI imagery and various pixel-based classifiers, and enhanced this approach by integrating fully polarimetric synthetic aperture radar data, resulting in more accurate mapping of these agricultural fields. Building on this, the present study conducted field experiments from 2021 to 2022, utilizing a method based on supervised classifiers to monitor the degradation area of biodegradable mulch film. This approach is impervious to external interferences, enabling accurate identification of the biodegradable mulch film rupture area. Under these conditions, we can precisely determine the evaporation and transpiration processes in cotton fields under varying biodegradable mulch film coverage areas, effectively guiding irrigation practices and preventing the disconnect between biodegradable mulch application and cotton production. Moreover, it reveals the compensatory mechanisms by which increased irrigation depth mitigates decreases in the soil moisture and cotton yield under biodegradable mulch film coverage. At an irrigation depth of 495 mm, biodegradable mulch film can effectively replace traditional plastic mulch, providing a theoretical foundation for the green and sustainable development of cotton production in arid regions of Northwest China.

## 5 Conclusion

This study employed the color channel threshold segmentation method to realize the accurate and quantitative monitoring of the process of biodegradable mulch film damage, and overcame the limitations of the traditional visual method, which is highly subjective and the serious lag of the weight loss method. Under the coverage of biodegradable mulch film, for every 1% increase in the damage rate, soil evaporation increased by 0.34 mm·d^−1^ (R**^2^ = **0.6027, n = 1613, and p = 0.028), revealing the direct driving mechanism of degradation processes on water loss. More importantly, the study demonstrates that under typical arid and semi-arid ecological conditions, implementing an optimized irrigation regime of 495 mm can effectively compensate for water stress caused by plastic mulch degradation. This approach increases soil moisture content by 5.29–15.37% and significantly elevates the proportion of crop water use dominated by transpiration efficiency. Ultimately, it drives cotton yield increases of 5.50–14.55%. This achievement provides a systematic solution combining precise monitoring methods with quantitative irrigation strategies, addressing the dual challenges of agricultural white pollution and efficient water resource utilization in arid and semi-arid regions.

## Data Availability

The raw data supporting the conclusions of this article will be made available by the authors, without undue reservation.

## References

[B1] Allen R. G. Pereira L. S. Raes D. Smith M. (1998). “ Crop evapotranspiration,” in Guidelines for Computing Crop Water Requirements – FAO Irrigation and Drainage ( FAO, Rome), 56.

[B2] Bian J. Hu X. Shi L. Min L. Zhang Y. Shen Y. . (2024). Evapotranspiration partitioning by integrating eddy covariance, micro-lysimeter and unmanned aerial vehicle observations: a case study in the North China Plain. Agric. Water Manage. 295, 108735. doi: 10.1016/j.agwat.2024.108735

[B3] Brodhagen M. Peyron M. Miles C. Inglis D. A. (2015). Biodegradable plastic agricultural mulches and key features of microbial degradation. Appl. Microbiol. Biotechnol. 99, 1039–1056. doi: 10.1007/s00253-014-6267-5, PMID: 25487893

[B4] Burato A. Fichera D. Cornali S. Reggiani R. Ronga D. (2025). Soil-biodegradable mulching films improve yield, water productivity, and profitability in organic processing tomato. Ital. J. Agron. 20, 100035. doi: 10.1016/j.ijagro.2025.100035

[B5] Campbell D. N. Na C. I. Rowland D. L. Schnell R. W. Ferrell J. A. Wilkie A. C. (2015). Development of a regional specific crop coefficient (Kc) for castor (Ricinus communis L.) in Florida, USA by using the sap flow method. Ind. Crops Prod. 74, 465–471. doi: 10.1016/j.indcrop.2015.04.006

[B6] Chai Y. Chai Q. Yang C. Chen Y. Li R. Li Y. . (2022). Plastic film mulching increases yield, water productivity, and net income of rain-fed winter wheat compared with no mulching in semiarid Northwest China. Agric. Water Manage. 262, 107420. doi: 10.1016/j.agwat.2021.107420

[B7] Chen N. Li X. Shi H. Hu Q. Zhang Y. Leng X. (2021a). Effect of biodegradable film mulching on crop yield, soil microbial and enzymatic activities, and optimal levels of irrigation and nitrogen fertilizer for the Zea mays crops in arid region. Sci. Total Environ. 776, 145970. doi: 10.1016/j.scitotenv.2021.145970, PMID: 33647668

[B8] Chen N. Li X. Shi H. Yan J. Hu Q. Zhang Y. (2021b). Modeling maize evapotranspiration and associated processes under biodegradable film mulching in an arid dripped field. Agric. For. Meteorol. 297, 108247. doi: 10.1016/j.agrformet.2020.108247

[B9] Comas L. H. Trout T. J. DeJonge K. C. Zhang H. Gleason S. M. (2019). Water productivity under strategic growth stage-based deficit irrigation in maize. Agric. Water Manage. 212, 433–440. doi: 10.1016/j.agwat.2018.07.015

[B10] Cui Y. Tian Z. Zhang X. Muhammad A. Han H. Jiang D. . (2015). Effect of water deficit during vegetative growth periods on post-anthesis photosynthetic capacity and grain yield in winter wheat (Triticum aestivum L.). Acta Physiol. Plant 37, 1–10. doi: 10.1007/s11738-015-1944-2

[B11] Deng L. Yu Y. Zhang H. Wang Q. Yu R. (2019). The effects of biodegradable mulch film on the growth, yield, and water use efficiency of cotton and maize in an arid region. Sustainability 11, 7039. doi: 10.3390/su11247039

[B12] De Sadeleer I. Woodhouse A. (2024). Environmental impact of biodegradable and non-biodegradable agricultural mulch film: A case study for Nordic conditions. Int. J. Life Cycle Assess. 29, 275–290. doi: 10.1007/s11367-023-02253-y

[B13] Ding R. Kang S. Zhang Y. Hao X. Tong L. Du T. (2013). Partitioning evapotranspiration into soil evaporation and transpiration using a modified dual crop coefficient model in irrigated maize field with ground-mulching. Agric. Water Manage. 127, 85–96. doi: 10.1016/j.agwat.2013.05.018

[B14] Evett S. R. Warrick A. W. Matthias A. D. (1995). Wall material and cap effects on microlysimeter temperatures and evaporation. Soil Sci. Soc Am. J. 59, 329–336. doi: 10.2136/sssaj1995.03615995005900020009x

[B15] Fan Y. Ding R. Kang S. Hao X. Du T. Tong L. . (2017). Plastic mulch decreases available energy and evapotranspiration and improves yield and water use efficiency in an irrigated maize cropland. Agric. Water Manage. 179, 122–131. doi: 10.1016/j.agwat.2016.08.019

[B16] Fang H. Li Y. Gu X. Chen P. Li Y. (2022). Root characteristics, utilization of water and nitrogen, and yield of maize under biodegradable film mulching and nitrogen application. Agric. Water Manage. 262, 107392. doi: 10.1016/j.agwat.2021.107392

[B17] Fang H. Li Y. Gu X. Yu M. Du Y. Chen P. . (2021). Evapotranspiration partitioning, water use efficiency, and maize yield under different film mulching and nitrogen application in northwest China. Field Crops Res. 264, 108103. doi: 10.1016/j.fcr.2021.108103

[B18] Fernández J. E. Alcon F. Diaz-Espejo A. Hernandez-Santana V. Cuevas M. V. (2020). Water use indicators and economic analysis for on-farm irrigation decision: A case study of a super high density olive tree orchard. Agric. Water Manage 237, 106074. doi: 10.1016/j.agwat.2020.106074

[B19] French A. N. Hunsaker D. J. Bounoua L. Karnieli A. Luckett W. E. Strand R. (2018). Remote sensing of evapotranspiration over the central Arizona irrigation and drainage district, USA. Agronomy 8, 278. doi: 10.3390/agronomy8120278

[B20] Gao H. Yan C. Liu Q. Ding W. Chen B. Li Z. (2019). Effects of plastic mulching and plastic residue on agricultural production: A meta-analysis. Sci. Total Environ. 651, 484–492. doi: 10.1016/j.scitotenv.2018.09.105, PMID: 30243168

[B21] Gong Z. Gao F. Chang X. Hu T. Li Y. (2024). A review of interactions between irrigation and evapotranspiration. Ecol. Indic. 169, 112870. doi: 10.1016/j.ecolind.2024.112870

[B22] Gu X. Cai H. Fang H. Chen P. Li Y. Li Y. (2021). Soil hydro-thermal characteristics, maize yield and water use efficiency as affected by different biodegradable film mulching patterns in a rain-fed semi-arid area of China. Agric. Water Manage. 245, 106560. doi: 10.1016/j.agwat.2020.106560

[B23] Gu X. B. Li Y. N. Du Y. D. (2016). Continuous ridges with film mulching improve soil water content, root growth, seed yield and water use efficiency of winter oilseed rape. Ind. Crops Prod. 85, 139–148. doi: 10.1016/j.indcrop.2016.02.056

[B24] Guo H. Li S. (2024). A review of drip irrigation’s effect on water, carbon fluxes, and crop growth in farmland. Water 16, 2206. doi: 10.3390/w16152206

[B25] Ha S. Chen Z. li F. Hu Y. (2020). Mapping plastic-mulched farmland by coupling optical and synthetic aperture radar remote sensing. Int. J. Remote Sens 41, 7757–7778. doi: 10.1080/01431161.2020.1763510

[B26] Ham J. M. Heilman J. L. Lascano R. J. (1990). Determination of soil water evaporation and transpiration from energy balance and stem flow measurements. Agric. For. Meteorol. 52, 287–301. doi: 10.1016/0168-1923(90)90087-M

[B27] Han Y. Zhang J. Chen P. Li H. Li W. Liu J. . (2024). Biochar improves water and nitrogen use efficiency of cotton under mulched drip irrigation in arid regions. Ind. Crops Prod. 222, 119830. doi: 10.1016/j.indcrop.2024.119830

[B28] He H. Wang Z. Guo L. Zheng X. Zhang J. Li W. . (2018). Distribution characteristics of residual film over a cotton field under long-term film mulching and drip irrigation in an oasis agroecosystem. Soil till. Res. 180, 194–203. doi: 10.1016/j.still.2018.03.013

[B29] Hou X. Fan J. Zhang F. Hu W. Yan F. Xiao C. . (2022). Determing water use and crop coefficients of drip-irrigated cotton in south Xinjiang of China under various irrigation amounts. Ind. Crops Prod. 176, 114376. doi: 10.1016/j.indcrop.2021.114376

[B30] Irmak S. Skaggs K. E. Chatterjee S. (2014). A review of the Bowen ratio surface energy balance method for quantifying evapotranspiration and other energy fluxes. Trans. ASABE 57, 1657–1674. doi: 10.13031/trans.57.10686

[B31] Isaev S. Khasanov S. Ashirov Y. Karabaeva T. Gofirov A. (2021). Effect of water and resource saving technologies of cotton growing on cotton yield. E3S Web Conferences 244, 2012. doi: 10.1051/e3sconf/202124402012

[B32] Kasirajan S. Ngouajio M. (2012). Polyethylene and biodegradable mulches for agricultural applications: a review. Agron. Sustain. Dev. 32, 501–529. doi: 10.1007/s13593-011-0068-3

[B33] Li F. Bai J. Y. Zhang M. Y. Zhang R. Y. (2022). Yield estimation of high-density cotton fields using low-altitude UAV imaging and deep learning. Plant Methods 18, 55. doi: 10.1186/s13007-022-00881-3, PMID: 35477580 PMC9044671

[B34] Li N. Shi X. Zhang H. Shi F. Zhang H. Wang J. (2024). Optimizing irrigation strategies to improve the soil microenvironment and enhance cotton water productivity under deep drip irrigation. Agric. Water Manage 305, 109095. doi: 10.1016/j.agwat.2024.109095

[B35] Li Z. Zhang F. Ma Y. Wan S. Han Y. Chen G. . (2024). Rational optimization of irrigation regimes for drip-irrigated cotton fields without mulch can alleviate the problem of residual film contamination in arid zones. Ind. Crops Prod. 221, 119430. doi: 10.1016/j.indcrop.2024.119430

[B36] Liebhard G. Klik A. Neugschwandtner R. W. Nolz R. (2022). Effects of tillage systems on soil water distribution, crop development, and evaporation and transpiration rates of soybean. Agric. Water Manage 269, 107719. doi: 10.1016/j.agwat.2022.107719

[B37] Liu X. He B. Yi X. Zhang L. Han F. (2016). The soil water dynamics and hydraulic processes of crops with plastic film mulching in terraced dryland fields on the Loess Plateau. Environ. Earth Sci. 75, 1–16. doi: 10.1007/s12665-016-5670-x

[B38] Liu Q. Wang Y. Liu J. Liu X. Dong Y. Huang X. . (2022). Degradability and properties of PBAT-based biodegradable mulch films in field and their effects on cotton planting. Polymers 14, 3157. doi: 10.3390/polym14153157, PMID: 35956671 PMC9371060

[B39] Lu S. Zhu G. Qiu D. Li R. Jiao Y. Meng G. . (2025). Optimizing irrigation in arid irrigated farmlands based on soil water movement processes: knowledge from water isotope data. Geoderma 460, 117440. doi: 10.1016/j.geoderma.2025.117440

[B40] Oberholzer S. Prasuhn V. Hund A. (2017). Crop water use under Swiss pedoclimatic conditions–Evaluation of lysimeter data covering a seven-year period. Field Crops Res. 211, 48–65. doi: 10.1016/j.fcr.2017.06.003

[B41] Qi H. Zhao G. Wang Y. Liu J. Gengshao (2021). Research progress on pollution hazards and prevention measures of residual film in cotton field in China. Cotton Sci. 33, 169–179. doi: 10.11963/1002-7807

[B42] Qin S. Li S. Yang K. Hu K. (2018). Can plastic mulch save water at night in irrigated croplands? J. Hydrol. 564, 667–681. doi: 10.1016/j.jhydrol.2018.07.050

[B43] Sadras V. Whitfield D. Connor D. (1991). Regulation of evapotranspiration, and its partitioning between transpiration and soil evaporation by sunflower crops: a comparison between hybrids of different stature. Field Crops Res. 28, 17–37. doi: 10.1016/0378-4290(91)90071-3

[B44] Scott R. L. (2010). Using watershed water balance to evaluate the accuracy of eddy covariance evaporation measurements for three semiarid ecosystems. Agric. For. Meteorol. 150, 219–225. doi: 10.1016/j.agrformet.2009.11.002

[B45] Sellami M. H. Di Mola I. Ottaiano L. Cozzolino E. del Piano L. Mori M. (2024). Evaluation of biodegradable mulch films on melon production and quality under mediterranean field conditions. Agronomy 14, 2075. doi: 10.3390/agronomy14092075

[B46] Sharma R. Bhardwaj S. (2017). Effect of mulching on soil and water conservation-A review. Agric. Syst. 38, 311–315. doi: 10.18805/ag.R-1732

[B47] Song Z. Zhao L. Bi J. Tang Q. Wang G. Li Y. (2024). Classification of degradable mulch films and their promotional effects and limitations on agricultural production. Agriculture 14, 1235. doi: 10.3390/agriculture14081235

[B48] Su J. Liu C. Coombes M. Hu X. Chen W. (2018). Wheat yellow rust monitoring by learning from multispectral UAV aerial imagery. Comput. Electron. Agric. 155, 157–166. doi: 10.1016/j.compag.2018.10.017

[B49] Sun D. Li H. Wang E. He W. Hao W. Yan C. . (2020). An overview of the use of plastic-film mulching in China to increase crop yield and water-use efficiency. Natl. Sci. Rev. 7, 1523–1526. doi: 10.1093/nsr/nwaa146, PMID: 34691485 PMC8290964

[B50] Tan Y. Chai Q. Li G. Hu F. Yu A. Zhao C. . (2022). No-till and nitrogen fertilizer reduction improve nitrogen translocation and productivity of spring wheat (Triticum aestivum L.) via promotion of plant transpiration. Front. Plant Sci. 13. doi: 10.3389/fpls.2022.988211, PMID: 36119600 PMC9478441

[B51] Touchaleaume F. Martin-Closas L. Angellier-Coussy H. Chevillard A. Cesar G. Gontard N. . (2016). Performance and environmental impact of biodegradable polymers as agricultural mulching films. Chemosphere 144, 433–439. doi: 10.1016/j.chemosphere.2015.09.006, PMID: 26386433

[B52] Unkovich M. Baldock J. Farquharson R. (2018). Field measurements of bare soil evaporation and crop transpiration, and transpiration efficiency, for rainfed grain crops in Australia–A review. Agric. Water Manage 205, 72–80. doi: 10.1016/j.agwat.2018.04.016

[B53] Wang Z. Wu Q. Fan B. Zhang J. Li W. Zheng X. (2019). Testing biodegradable films as alternatives to plastic films in enhancing cotton (Gossypium hirsutum L.) yield under mulched drip irrigation. Soil Tillage Res. 192, 196–205. doi: 10.1016/j.still.2019.05.004

[B54] Wang F. Wang K. Sun X. Long B. Zhong L. Li F. . (2024). Polybutylene adipate terephthalic acid (PBAT) biodegradable mulching films effectively affect the nutrition metabolism and growth of chewing cane compared to polyethylene mulching films. Ind. Crops Prod. 222, 119958. doi: 10.1016/j.indcrop.2024.119958

[B55] Wang Y. Yin W. Hu F. Fan Z. Zhao C. Yu A. . (2022). Interspecies interaction intensity influences water consumption in wheat–maize intercropping by regulating root length density. Crop Sci. 62, 441–454. doi: 10.1002/csc2.20639

[B56] Wang J. Zhou Q. Chen R. Wang Z. (2025). Rationale saline-water irrigation also serves as enhancing soil aggregate stability, regulating carbon emissions, and improving water use efficiency in oasis cotton fields. Ind. Crops Prod. 223, 120144. doi: 10.1016/j.indcrop.2024.120144

[B57] Wei X. Zheng S. Chen D. Qiu L. (2025). Estimation of evapotranspiration and its components of drip-irrigated grapevine under greenhouse conditions in cold Northeast China. Irrig. Sci. 43, 755–772. doi: 10.1007/s00271-025-01016-x

[B58] Wu Y. Li Q. Zhong X. Gong D. Liu X. (2025). Effect of different data quality control on evapotranspiration of winter wheat with Bowen ratio method. Agric. Water Manage 311, 109379. doi: 10.1016/j.agwat.2025.109379

[B59] Xia X. Hu D. Liu X. Yue L. Ma B. Chen Y. . (2024). Partitioning evapotranspiration of Camellia oleifera during the growing season based on the Penman-Monteith model combined with the micro-lysimeter and stable isotope methods. Agric. Water Manage 297, 108831. doi: 10.1016/j.agwat.2024.108831

[B60] Xiong L. Li Z. Shah F. Wang P. Yuan Q. Wu W. (2024). Biodegradable mulch film enhances the environmental sustainability compared with traditional polyethylene film from multidimensional perspectives. Chem. Eng. J. 492, 152219. doi: 10.1016/j.cej.2024.152219

[B61] Yang J. Zhai Z. Li Y. Duan H. Cai F. Lv J. . (2024). Design and research of residual film pollution monitoring system based on UAV. Comput. Electron. Agric. 217, 108608. doi: 10.1016/j.compag.2023.108608

[B62] Yang C. Zhao Y. Long B. Wang F. Li F. Xie D. (2023). Biodegradable mulch films improve yield of winter potatoes through effects on soil properties and nutrients. Ecotox. Environ. safe. 264, 115402. doi: 10.1016/j.ecoenv.2023.115402, PMID: 37634481

[B63] Yin M. Li Y. Fang H. Chen P. (2019). Biodegradable mulching film with an optimum degradation rate improves soil environment and enhances maize growth. Agric. Water Manage 216, 127–137. doi: 10.1016/j.agwat.2019.02.004

[B64] Zhang X. Luo C. Ren H. Dai R. Mburu D. Kavag L. . (2021). Fully biodegradable film to boost rainfed maize (Zea mays L.) production insemiarid Kenya: An environmentally friendly perspective. Eur. J. Agron. 119, 126124. doi: 10.1016/j.eja.2020.126124

[B65] Zhang H. Wang D. Zhang X. Wang Y. Liu H. Tang Q. . (2025). Response of the soil hydrothermal environment and cotton yield to different irrigation quotas under biodegradable mulch film in oasis cotton fields: a three-year study. Front. Plant Sci. 16. doi: 10.3389/fpls.2025.1521635, PMID: 40190651 PMC11968355

[B66] Zhao Z. Wu H. Jin T. Liu H. Men J. Cai G. . (2023). Biodegradable mulch films significantly affected rhizosphere microbial communities and increased peanut yield. Sci. Total Environ. 871, 162034. doi: 10.1016/j.scitotenv.2023.162034, PMID: 36754316

[B67] Zhao X. Yuan Y. T. Song M. D. Ding Y. Lin F. F. Liang D. . (2019). Use of unmanned aerial vehicle imagery and deep learning UNet to extract rice lodging. Sensors 19, 3859. doi: 10.3390/s19183859, PMID: 31500150 PMC6766838

[B68] Zhou L. Li F. Jin S. Song Y. (2009). How two ridges and the furrow mulched with plastic film affect soil water, soil temperature and yield of maize on the semiarid Loess Plateau of China. Field Crops Res. 113, 41–47. doi: 10.1016/j.fcr.2009.04.005

[B69] Zong R. Wang Z. Zhang J. Li W. (2021). The response of photosynthetic capacity and yield of cotton to various mulching practices under drip irrigation in Northwest China. Agric. Water Manage 249, 106814. doi: 10.1016/j.agwat.2021.106814

[B70] Zribi W. Aragüés R. Medina E. Faci J. M. (2015). Efficiency of inorganic and organic mulching materials for soil evaporation control. Soil Till. Res. 148, 40–45. doi: 10.1016/j.still.2014.12.003

[B71] Zuo W. Wu B. Wang Y. Xu S. Tian J. Jiu X. . (2023). Optimal planting pattern of cotton is regulated by irrigation amount under mulch drip irrigation. Front. Plant Sci. 14. doi: 10.3389/fpls.2023.1158329, PMID: 37324720 PMC10265678

